# Molecular Interplay between AURKA and SPOP Dictates CRPC Pathogenesis via Androgen Receptor

**DOI:** 10.3390/cancers12113247

**Published:** 2020-11-04

**Authors:** Kumar Nikhil, Mohini Kamra, Asif Raza, Hanan S. Haymour, Kavita Shah

**Affiliations:** Department of Chemistry and Purdue University Center for Cancer Research, Purdue University, 560 Oval Drive, West Lafayette, IN 47907, USA; knikhil@purdue.edu (K.N.); mkamra@purdue.edu (M.K.); razaasif.biotech@gmail.com (A.R.); hhaymour@purdue.edu (H.S.H.)

**Keywords:** Aurora kinase A (AURKA), SPOP, epithelial to mesenchymal transition, therapy, prostate cancer, CRPC

## Abstract

**Simple Summary:**

Prostate cancer is the most common type of cancer in men. Early stage prostate cancer is treatable. However, remission eventually occurs in vast majority of patients, giving rise to castration-resistant prostate cancer (CRPC), which is incurable with current therapies. Aurora A (AURKA) is overexpressed in all stages of prostate cancer including CRPC. AURKA inhibition has shown efficacy in reducing cancer burden in clinical trials, however, no AURKA inhibitor has been approved by the FDA, primarily because AURKA inhibition is toxic to normal cells. Thus, identification of downstream targets of AURKA provides an alternative approach to regulate AURKA-mediated malignancy. We identified a tumor suppressor protein named SPOP as AURKA target. SPOP is rendered ineffective in prostate cancer by genomic mutations promoting cancer. We show that AURKA degrades SPOP, which promotes malignancy and drug-resistance. Thus, AURKA inhibition provides a powerful tool to retain SPOP, thereby treating the disease and inhibiting its progression.

**Abstract:**

SPOP, an adaptor protein for E3 ubiquitin ligase can function as a tumor-suppressor or a tumor-enhancer. In castration-resistant prostate cancer (CRPC), it inhibits tumorigenesis by degrading many oncogenic targets, including androgen receptor (AR). Expectedly, SPOP is the most commonly mutated gene in CRPC (15%), which closely correlates with poor prognosis. Importantly, 85% of tumors that retain wild-type SPOP show reduced protein levels, indicating that SPOP downregulation is an essential step in CRPC progression. However, the underlying molecular mechanism remains unknown. This study uncovered the first mechanism of SPOP regulation in any type of cancer. We identified SPOP as a direct substrate of Aurora A (AURKA) using an innovative technique. AURKA directly phosphorylates SPOP at three sites, causing its ubiquitylation. SPOP degradation drives highly aggressive oncogenic phenotypes in cells and in vivo including stabilizing AR, ARv7 and c-Myc. Further, SPOP degrades AURKA via a feedback loop. SPOP upregulation is one of the mechanisms by which enzalutamide exerts its efficacy. Consequently, phospho-resistant SPOP fully abrogates tumorigenesis and EMT in vivo, and renders CRPC cells sensitive to enzalutamide. While genomic mutations of SPOP can be treated with gene therapy, identification of AURKA as an upstream regulator of SPOP provides a powerful opportunity for retaining WT-SPOP in a vast majority of CRPC patients using AURKA inhibitors ± enzalutamide, thereby treating the disease and inhibiting its progression.

## 1. Introduction

Aurora kinase A (AURKA) is a serine/threonine protein kinase critical for mitotic spindle formation and chromosome segregation [1]. Aberrant expression of AURKA supersedes the mitotic spindle checkpoint and induces aneuploidy, leading to genetic transformation and chemoresistance [2]. AURKA promotes cancer by inhibiting apoptosis and augmenting cell survival, cell cycle progression, tumorigenicity, epithelial-mesenchymal transition (EMT) and stem cell-like properties. Overexpression of AURKA is reported in numerous tumors including melanoma, leukemia, pancreatic, ovarian, gastrointestinal, esophageal kidney, colon, breast and prostate cancer (PCa) [3]. AURKA inhibition or depletion inhibits apoptosis and attenuates tumor growth in many cancers [4]. More than a dozen AURKA inhibitors including alisertib, MK-5108, ENMD-2076 and danusertib are in different phases of clinical trials [5]. In a Phase II clinical trial, alisertib showed significant clinical benefit in a subset of patients suffering from CRPC and neuroendocrine PCa [6]. Nevertheless, FDA has approved no AURKA inhibitor yet, primarily due to the toxicity associated with collateral AURKA inhibition in normal proliferating cells. Thus, an alternative approach to regulate AURKA is to identify and modulate its downstream targets in different cancers. Several downstream targets of AURKA have been identified including BRCA1, N-myc, GSK3β, LIMK2, TWIST1 and ALDH1A1 in various cancers [7,8,9,10,11,12,13]. We recently reported YBX1 as an oncogenic target and regulator of AURKA in castration resistant prostate cancer (CRPC) [14].

AURKA levels are considerably increased in prostatic intraepithelial neoplasia (PIN) lesions and prostate tumors as compared to its levels in non-neoplastic specimens [15]. AURKA is also abundantly expressed in androgen receptor (AR)-positive CRPC, as AR upregulates AURKA transcription [16]. In turn, AURKA promotes the transcription of ARv7, a spliced form of AR, but not of full-length AR [17]. AR is overexpressed in a vast majority of CRPC cases and remains the most critical driver in both hormone-naïve PCa and CRPC [18]. Furthermore, prolonged inhibition of AR signaling using androgen-signaling inhibitors such as enzalutamide, triggers compensatory adaptive responses including upregulation of spliced isoforms of AR including ARv7, which drives acquired enzalutamide resistance in CRPC. Interestingly, AR gene amplification occurs only in 10% of the cases, indicating the existence of additional guiding mechanisms at post-transcriptional, transcriptional and post-translational stages [19]. Previously, we uncovered that AURKA stabilizes AR by upregulating YBX1 [14]. In the present study, we have discovered another fatal mechanism of AR and ARv7 upregulation at a post-translational level triggered by AURKA. We recently identified Speckle-type POZ (pox virus and zinc finger) protein (SPOP) as a direct target of AURKA using an innovative chemical-genetic screen [20]. SPOP, an E3 ubiquitin ligase adaptor, can function either as a tumor suppressor or a tumor promoter. In PCa, SPOP acts as a tumor suppressor as it binds and degrades several oncogenic targets including AR [21,22]. As expected, SPOP is the most altered gene in PCa (~15%), which abrogates its binding to its substrates, thereby rendering it ineffective, which correlates with poor prognosis in patients [23]. These mutations are specific to PCa and are rarely detected in other cancers. Intriguingly, a vast majority (~94%) of the remaining 85% tumors which possess wild-type (WT) SPOP exhibit very low levels of the protein, indicating that the modulation of SPOP is an essential step in CRPC progression [24,25]. However, the molecular mechanism of SPOP’s downregulation remains unknown. Although oncogenic phenotypes caused by SPOP mutants (SPOP^MT^) have never been correlated with AURKA overexpression phenotypes in cells or in vivo, nevertheless, phenotypes caused by SPOP^MT^ in PCa are analogous to aggressive oncogenic phenotypes caused by AURKA overexpression. Furthermore, AURKA is often known to modulate its substrate levels upon phosphorylation. We have previously shown that AURKA upregulates LIMK2, TWIST1, ALDH1A1 and YBX1, but downregulates PHLDA1, via direct phosphorylation [11,12,13,14,20]. These findings led us to hypothesize that AURKA may be responsible for SPOP downregulation in CRPC via direct phosphorylation. This study reports the first post-translational regulation of SPOP triggered by AURKA in CRPC, which results in its robust downregulation. This, in turn, stabilizes AR, ARv7 and c-Myc, and promotes highly aggressive phenotypes including tumorigenesis, EMT and chemoresistance. 

## 2. Results

### 2.1. SPOP Is a Direct Substrate of AURKA and They Interact with Each Other in CRPC Cells

**Figure 1:**
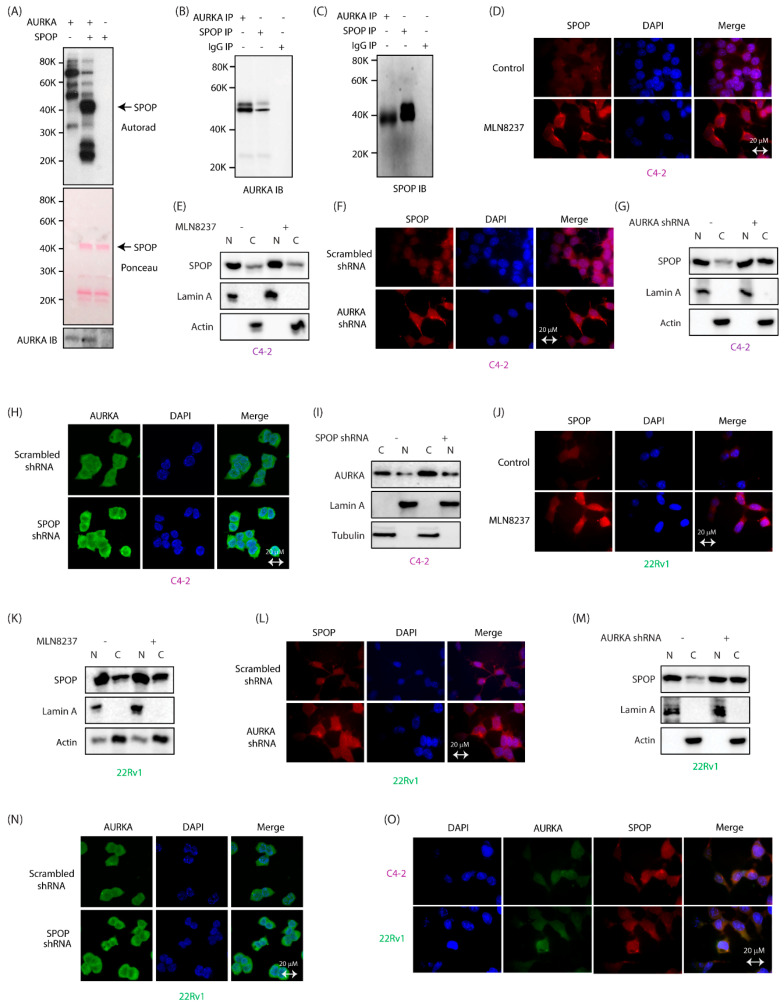
AURKA binds and phosphorylates SPOP and regulates its subcellular localization in CRPC cells. (**A**) SPOP is phosphorylated by AURKA in vitro. AURKA-TPX2 was incubated with [^32^P]ATP in a kinase buffer in lane 1. Lane 2 shows incubation of 6x-His-SPOP, AURKA-TPX2 and [^32^P]ATP in a kinase assay buffer for 30 min. Lane 3 shows 6x-His-SPOP incubated with [^32^P]ATP. (**B**) AURKA associates with SPOP in cells. SPOP immune complex was isolated from C4-2 cells, and AURKA binding analyzed (lane 2). AURKA IP (lane 1) was used as a positive control, and IgG IP (lane 3) was used as a negative control. (**C**) AURKA immune complex was isolated from C4-2 cells, and SPOP binding analyzed (lane 1). SPOP IP (lane 2) was used as a positive control, and IgG IP (lane 3) was used as a negative control. (**D**) AURKA inhibition using MLN8237 slightly alters the subcellular localization of SPOP in C4-2 cells. C4-2 cells were treated with 1 μM MLN8237 for 12 h, and subcellular localization of SPOP analyzed using SPOP-specific antibody (red). DAPI is shown in blue. (**E**) Subcellular fractionation of DMSO-treated and MLN8237-treated C4-2 cells do not show a significant change in SPOP’s residence upon AURKA inhibition. C4-2 cells were treated with DMSO or MLN8237 (1 μM) for 12 h, followed by fractionation. (**F**) AURKA knockdown increases the cytoplasmic localization of SPOP. C4-2 cells were treated with control or AURKA shRNA for 30 h, fixed and stained with SPOP antibody (red) and DAPI (blue). >100 cells were analyzed from multiple random frames. (**G**) Subcellular fractionation of scrambled shRNA-treated and AURKA shRNA-treated C4-2 cells confirm increased cytoplasmic localization of SPOP upon AURKA knockdown. C4-2 cells were treated with control or AURKA shRNA for 30 h, followed by fractionation. (**H**) SPOP depletion does not affect the subcellular location of AURKA in C4-2 cells. C4-2 cells were treated with either scrambled shRNA or SPOP shRNA for 30 h, fixed and stained with AURKA antibody (green) and DAPI (blue). (**I**) Subcellular fractionation of scrambled shRNA-treated and SPOP shRNA-treated C4-2 cells confirms no change in AURKA’s residence upon SPOP knockdown. C4-2 cells were treated with control or SPOP shRNA for 30 h, followed by fractionation. (**J**) AURKA inhibition using MLN8237 increases the cytoplasmic localization of SPOP in 22Rv1 cells. 22Rv1 cells were treated as described in 1D. (**K**) Subcellular fractionation of DMSO-treated and MLN8237-treated 22Rv1 cells show increased cytoplasmic residence of SPOP upon AURKA inhibition. 22Rv1 cells were treated as depicted in 1E. (**L**) AURKA depletion increases cytoplasmic residence of SPOP in 22Rv1 cells. 22Rv1 cells were treated with control or AURKA shRNA for 30 h, fixed and stained with SPOP antibody (red) or DAPI (blue). >100 cells were analyzed from multiple random frames. (**M**) Subcellular fractionation of scrambled shRNA-treated and AURKA shRNA-treated 22Rv1 cells confirm increased cytoplasmic localization of SPOP upon AURKA knockdown. (**N**) SPOP depletion does not affect the subcellular location of AURKA in 22Rv1 cells. 22Rv1 cells were treated with either scrambled shRNA or SPOP shRNA for 30 h, fixed and stained with AURKA antibody (green) and DAPI (blue). (**O**) AURKA and SPOP colocalize in the cytoplasm in C4-2 and 22Rv1 cells. C4-2 and 22Rv1 cells were fixed and stained with SPOP antibody (red), AURKA antibody (green) and DAPI (blue). >100 cells were analyzed from multiple random frames. Representative data are shown.

We generated 6x-His-tagged SPOP and subjected it to an in vitro phosphorylation assay with AURKA/TPX2. SPOP showed robust phosphorylation, validating it as a direct target of AURKA (Figure 1A, middle lane). This result prompted us to explore potential interaction of AURKA with SPOP in cells. We isolated endogenous SPOP via immunoprecipitation (IP) from C4-2 cells and analyzed potential AURKA binding. IgG IP and AURKA IP were used as negative and positive controls, respectively. AURKA was detected in SPOP immune complex (Figure 1B, middle lane). Similarly, AURKA IP detected SPOP in the immune complex (Figure 1C). These data confirm that both AURKA and SPOP physically associate with each other in C4-2 cells.

### 2.2. AURKA Increases Nuclear Localization of SPOP

We next investigated the subcellular localization of AURKA and SPOP in C4-2 cells. SPOP was primarily nuclear, with some cytoplasmic localization. In contrast, AURKA was mostly cytoplasmic with some nuclear localization. The AURKA inhibitor MLN8237 (alisertib) slightly increased cytoplasmic localization of SPOP (Figure 1D). To validate these results, subcellular fractionation was conducted, which did not show significant change in SPOP’s residence upon AURKA inhibition (Figure 1E and Figure S1E). In contrast, AURKA knockdown showed a robust increase in SPOP’s cytoplasmic localization in C4-2 cells, indicating that AURKA presumably uses both its kinase activity and protein-protein interactions to regulate SPOP’s residence in cells (Figure 1F,G, full gel images of 1G are shown in Figure S1G). We also investigated whether SPOP regulates AURKA’s subcellular localization. SPOP knockdown did not change AURKA localization, as shown by immunofluorescence and confirmed using subcellular fractionation (Figure 1H,I and Figure S1I). Analogous results were obtained in 22Rv1 cells where both AURKA inhibition and knockdown resulted in enhanced cytoplasmic localization of SPOP, subsequently confirmed using subcellular fractionation (Figure 1J–M, and Figure S1K). In contrast, SPOP depletion did not affect AURKA subcellular localization (Figure 1N). As AURKA and SPOP directly interact with each other (Figure 1B,C), we investigated whether they interact with each other in the cytoplasm or nucleus. Our data show that AURKA and SPOP co-localize mainly in the cytoplasm in both C4-2 and 22Rv1 cells (Figure 1O).

### 2.3. AURKA Regulates SPOP Post-Translationally, But Not at mRNA Levels

**Figure 2:**
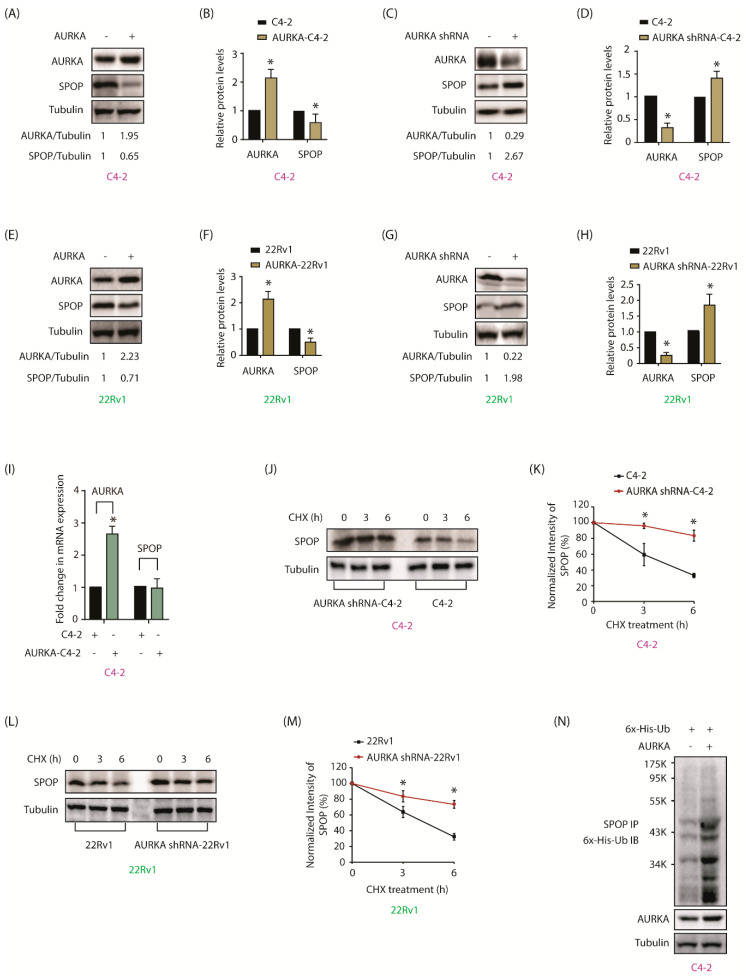
AURKA negatively regulates SPOP by degrading it in CRPC cells. (**A**) AURKA negatively regulates SPOP in C4-2 cells. (**B**) Histogram showing change in SPOP levels with AURKA overexpression. The data are presented as mean ± SEM obtained from three independent experiments. * *p* < 0.05 vs. C4-2 control cells. (**C**) AURKA silencing increases SPOP levels in C4-2 cells. C4-2 cells were infected with either scrambled shRNA lentivirus or AURKA shRNA lentivirus, followed by IB. (**D**) Histogram shows change in SPOP levels with AURKA knockdown. The data are presented as mean ± SEM obtained from three independent experiments. * *p* < 0.05 vs. C4-2 control cells. (**E**) Overexpression of AURKA decreased the levels of SPOP protein in 22Rv1 cells. (**F**) Histogram shows change in SPOP levels with AURKA overexpression. (**G**) AURKA silencing increases SPOP protein levels in 22Rv1 cells. (**H**) Histogram shows change in SPOP levels with AURKA knockdown. (**I**) AURKA overexpression does not affect SPOP mRNA levels. C4-2 cells were infected with AURKA or vector control retrovirus followed by RT-qPCR for mRNA expression. The data are presented as mean ± SEM obtained from three independent experiments. * *p* < 0.05 vs. C4-2 control cells. (**J**) AURKA increases SPOP degradation rate in C4-2 cells. C4-2 and AURKA-shRNA-C4-2 cells were treated with cycloheximide (10 μM) for 3 and 6 h and SPOP levels analyzed. The data are presented as mean ± SEM obtained from three independent experiments. * *p* < 0.05 vs. C4-2 control cells. (**K**) Graphical representation of SPOP degradation rate in C4-2 cells. (**L**) AURKA increases SPOP degradation rate in 22Rv1 cells. The data are presented as mean ± SEM obtained from three independent experiments. * *p* <0.05 vs. 22Rv1 control cells. (**M**) Graphical representation of SPOP degradation rate in 22Rv1 cells. (**N**) AURKA degrades SPOP by enhancing its ubiquitylation. C4-2 cells were co-infected with 6x-His-ubiquitin and AURKA retrovirus for 30 h followed by MG132 treatment for 12 h. SPOP was immunoprecipitated and ubiquitylation analyzed using 6x-His antibody. Each experiment was done at least three independent times and representative data are shown.

Interestingly, when AURKA was inhibited or depleted, SPOP immunofluorescence increased and vice versa, indicating that they might negatively regulate each other (Figure 1D,F,H,J,L,N). To test this hypothesis, AURKA was ectopically expressed, which diminished SPOP protein levels, while AURKA silencing using shRNA, significantly increased SPOP protein levels in C4-2 cells (Figure 2A,C, and Figure S2A,C). Figure 2B,D shows changes in SPOP protein levels in AURKA-C4-2 cells from three independent experiments. To confirm whether this change occurred in other CRPC cells, AURKA was similarly overexpressed and knocked-down in 22Rv1 cells and SPOP levels verified. These experiments showed analogous results (Figure 2E–H, and Figure S2E,G), signifying that AURKA negatively regulates SPOP in CRPC cells. To check whether this change was at the mRNA or protein level, SPOP mRNA levels were analyzed using RT-qPCR in control and AURKA-overexpressing cells. While there was a significant increase in AURKA mRNA levels (2.5-fold), no change in SPOP mRNA level was observed, suggesting that AURKA does not regulate SPOP mRNA levels in C4-2 cells (Figure 2I).

Therefore, we examined whether AURKA regulates SPOP stability. AURKA shRNA-C4-2 and C4-2 cells were treated with cycloheximide and half-life of SPOP was calculated. AURKA depletion stabilized SPOP protein and increased its half-life compared to its half-life in C4-2 cells (Figure 2J,K and Figure S2J). Analogous results were observed in 22Rv1 cells, where AURKA knockdown prolonged the half-life of SPOP (Figure 2L,M and Figure S2L). To confirm whether AURKA overexpression reduced SPOP due to ubiquitin-mediated degradation, we expressed 6x-His-ubiquitin in C4-2 and AURKA-C4-2 cells, which facilitated SPOP ubiquitylation, thereby confirming that AURKA degrades SPOP in a ubiquitin-dependent manner (Figure 2N).

### 2.4. SPOP Promotes AURKA Degradation in Response

**Figure 3:**
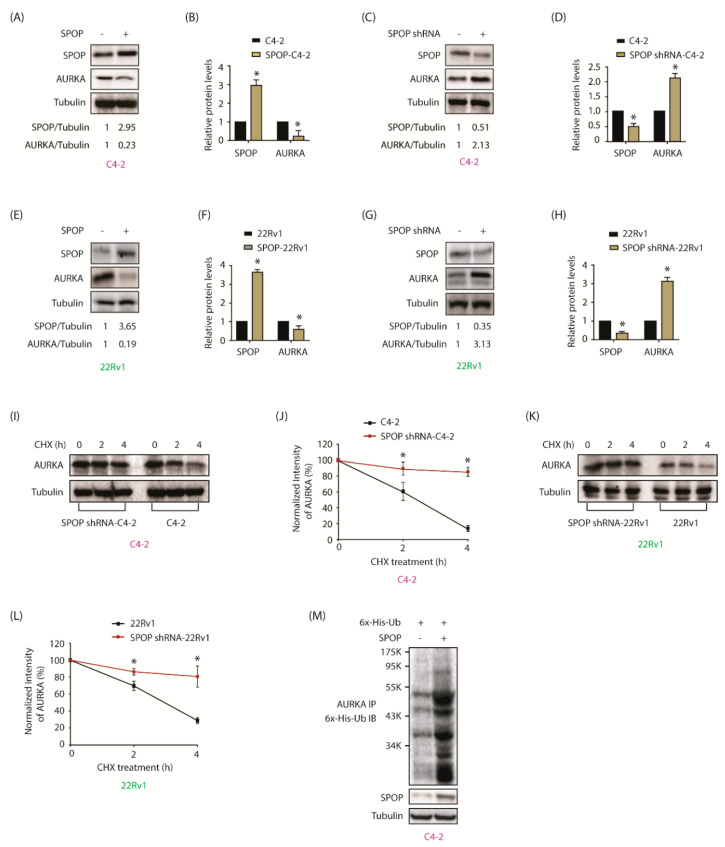
AURKA is targeted by SPOP in a reciprocal crosstalk. (**A**) SPOP inversely regulates AURKA protein levels in C4-2 cells. (**B**) Histogram shows decrease in AURKA level with SPOP overexpression. The data presented as mean ± SEM obtained from three independent experiments. * *p* < 0.05 vs. C4-2 control cells. (**C**) SPOP knockdown increases AURKA in C4-2 cells. (**D**) Histogram shows increase in AURKA levels with SPOP knockdown. The data are presented as mean ± SEM obtained from three independent experiments. * *p* < 0.05 vs. C4-2 control cells. (**E**) and (**F**) 22Rv1 cells showing change in AURKA protein levels with SPOP overexpression. (**G**) and (**H**) 22Rv1 cells showing change in AURKA protein levels after SPOP silencing. (**I**) SPOP decreases the half-life of AURKA. C4-2, and SPOP-shRNA-C4-2 were treated with 10 μM cycloheximide and protein lysates were collected at the indicated times for western blot analysis. (**J**) AURKA protein levels was quantified and plotted relative to the level at t = 0. (**K**) SPOP augments AURKA degradation in 22Rv1 cells. (**L**) Graphical representation of AURKA degradation rate in cells treated as in Figure 3K. (**M**) SPOP increases AURKA degradation by promoting its ubiquitylation. C4-2 cells were co-infected with 6x-His-ubiquitin (6x-His-Ub) along with SPOP retrovirus for 30 h, followed by MG132 treatment for 12 h. AURKA was immunoprecipitated and ubiquitylation analyzed using 6x-His antibody. Each experiment was done at least three independent times. Representative data are shown.

As our data revealed an increase in AURKA immunofluorescence upon SPOP knockdown, we examined whether SPOP regulates AURKA. AURKA levels decreased upon SPOP overexpression in C4-2 cells (Figure 3A,B and Figure S3A). Depletion of SPOP increased AURKA levels (Figure 3C,D and Figure S3C). 

Analogous results were obtained in 22Rv1 cells, leading us to conclude that SPOP negatively regulates AURKA in CRPC cells (Figure 3E–H and Figure S3E,G). Figure 3B,D,F,H show quantification of AURKA levels upon either SPOP overexpression or knockdown from three independent experiments. 

As SPOP ubiquitylates and degrades its target proteins [26], we examined whether SPOP controls AURKA stability by inhibiting protein synthesis in C4-2 and SPOP-depleted C4-2 cells. SPOP knockdown increased AURKA stability in C4-2 cells (Figure 3I,J and Figure S3J). Comparable results were observed in 22Rv1 cells, where SPOP knockdown prolonged the half-life of AURKA (Figure 3K,L and Figure S3L). Additionally, SPOP overexpression reduced AURKA levels due to ubiquitin-mediated degradation (Figure 3M), thereby confirming that SPOP ubiquitylates AURKA. These data demonstrate that AURKA is a new substrate of SPOP.

### 2.5. AURKA Phosphorylates SPOP at S33, T56 and S105

**Figure 4:**
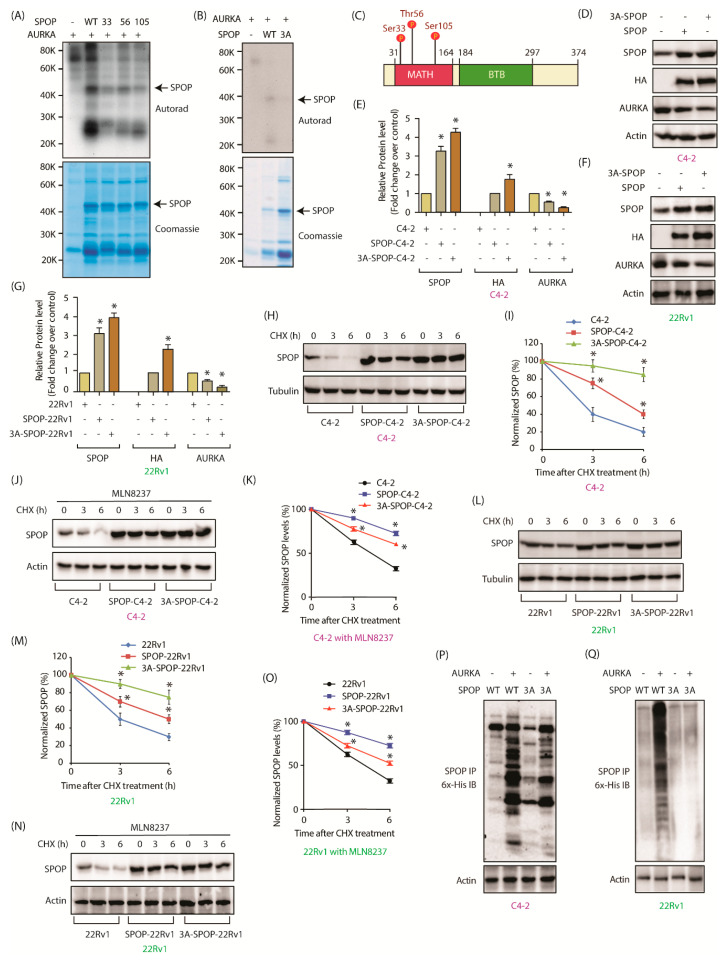
AURKA degrades SPOP by phosphorylation. (**A**) AURKA phosphorylates SPOP at S33, T56, and S105 positions. All three SPOP mutants were 6x-His-tagged. Kinase assay was conducted for 30 min. The top panel shows autoradiography and the bottom panel shows Coomassie staining. (**B**) AURKA phosphorylates SPOP only at S33, T56, and S105, as the corresponding 3A-phospho-resistant mutant did not show any phosphorylation. (**C**) S33, T56 and S105 are within the MATH domain of SPOP. (**D**) Phospho-resistant SPOP is expressed at relatively higher level compared to wild-type SPOP. C4-2 cells were infected with HA-tagged wild-type SPOP or 3A-SPOP retrovirus for 36 h followed by immunoblot analysis. (**E**) Quantification of wild type and mutant SPOP obtained from three independent experiments. The data are presented as mean ± SEM obtained from three independent experiments. * *p* < 0.05 vs. C4-2 control cells. (**F**) SPOP protein levels in 22Rv1 cells infected with HA-tagged wild-type SPOP or 3A-SPOP. (**G**) Quantification of SPOP obtained from three independent experiments in 22Rv1 cells. (**H**) Phospho-resistant SPOP has increased stability compared to wild type SPOP. SPOP levels were analyzed in C4-2, SPOP-C4-2 and 3A-SPOP-C4-2 cells treated with cycloheximide (10 μM) for 3 and 6 h. (**I**) Graphical representation of SPOP degradation profile in C4-2, SPOP-C4-2 and 3A-SPOP-C4-2 cells. (**J**) Inhibition of AURKA using MLN8237 does not affect the stability of 3A-SPOP mutant, but increases the stability of WT-SPOP in C4-2 cells. All three cell-types were treated with MLN8237 (1 μM) 12 h prior to 10 μM cycloheximide treatment. (**K**) Graphical representation of SPOP degradation profile in MLN8237-treated C4-2, SPOP-C4-2 and 3A-SPOP-C4-2 cells. (**L**) AURKA mediated phosphorylation of SPOP decreases its stability in 22Rv1 cells. 22Rv1, SPOP-22Rv1 and 3A-SPOP-22Rv1 cells were treated with cycloheximide for 3 h and 6 h, and SPOP levels analyzed. (**M**) Graphical representation of SPOP half-life in 22Rv1, SPOP-22Rv1 and 3A-SPOP-22Rv1 cells. (**N**) Inhibition of AURKA using MLN8237 does not affect the stability of 3A-SPOP mutant, but increases the stability of WT-SPOP in 22Rv1 cells. (**O**) Graphical representation of SPOP degradation profile in MLN8237-treated 22Rv1, SPOP-22Rv1 and 3A-SPOP-22Rv1 cells. (**P**) AURKA overexpression increased the ubiquitylation of WT-SPOP, but not of 3A-SPOP in C4-2 cells. WT-SPOP-C4-2 and 3A-SPOP-C4-2 cells were infected with 6x-His-Ubiquitin with or without AURKA retrovirus for 30 h, followed by MG132 treatment for 12 h. SPOP was immunoprecipitated using HA antibody and ubiquitylated SPOP was detected by 6x-His antibody. (**Q**) AURKA overexpression increased the ubiquitylation of WT-SPOP, but not of 3A-SPOP in 22Rv1 cells.

Based on AURKA’s preferred sequence specificity, we predicted three possible phosphorylation sites on SPOP-S33, T56 and S105. These were individually mutated to alanine. Recombinant S33A, T56A and S105A SPOP mutants were phosphorylated in vitro using AURKA, all of which showed reduced phosphorylation compared to the WT-SPOP (Figure 4A). Consequently, we generated a triple mutant of SPOP where all the three sites were mutated and performed an in vitro kinase assay using AURKA. The triple mutant (3A) showed almost no phosphorylation compared to WT-SPOP confirming that AURKA phosphorylates SPOP at S33, T56 and S105 positions only (Figure 4B). Interestingly, all three sites are present within the MATH domain (Figure 4C).

### 2.6. AURKA Decreases SPOP Stability via Phosphorylation 

Identification of SPOP phosphorylation sites led us to examine whether AURKA degrades SPOP via phosphorylation. WT and 3A-SPOP were transiently expressed in C4-2 and 22Rv1 cells, and SPOP levels analyzed.

3A-SPOP was expressed at comparatively higher levels with respect to wild-type in both C4-2 and 22Rv1 cells, with concomitant decrease in AURKA levels due to the feedback loop (Figure 4D–G and Figure S4D,F). The relative protein levels of WT-SPOP and 3A-SPOP obtained from three independent experiments are presented in Figure 4E,G. Subsequently, protein synthesis was inhibited in C4-2, WT-SPOP and 3A-SPOP expressing C4-2 cells using cycloheximide and the half-life of SPOP was measured. Phosphorylation affected SPOP protein stability as 3A-SPOP was found to be more stable compared to WT-SPOP in C4-2 cells (Figure 4H,I and Figure S4H). To further validate the role of AURKA in destabilizing SPOP, we examined the stability of SPOP in C4-2, SPOP-C4-2 and 3A-SPOP-C4-2 cells in the presence of MLN8237. While the stability of 3A-SPOP remained the same upon AURKA inhibition, both endogenous (in C4-2 cells) and ectopically expressed SPOP showed enhanced stability, thereby underscoring the impact of AURKA in SPOP degradation (Figure 4J,K and Figure S4J). Similar results were obtained in 22Rv1 cells, which confirmed enhanced stability of 3A-SPOP as compared to WT-SPOP (Figure 4L,M and Figure S4L). AURKA inhibition using MLN8237 increased the stability of WT-SPOP, but not 3A-SPOP, indicating that AURKA degrades SPOP by phosphorylating these sites in 22Rv1 cells as well (Figure 4N,O and Figure S4N). These results were further confirmed when ubiquitylation of wild-type SPOP was significantly higher as compared to ubiquitylation of 3A-SPOP cells in both cell types (Figure 4P,Q).

As our results showed that AURKA regulates the subcellular localization of SPOP (Figure 1D–G,J–M), we examined whether this process was phosphorylation-dependent. WT-SPOP showed predominantly nuclear localization with relatively faint cytoplasmic staining in both C4-2 and SPOP-C4-2 cells (Supplementary Figure S5A). In contrast, phospho-resistant 3A-SPOP was present more in the cytoplasm. Similar results were obtained in 22Rv1 cells, thereby confirming that AURKA promotes nuclear localization of SPOP via phosphorylation (Supplementary Figure S5B).

### 2.7. SPOP Inhibits Cell Proliferation, Colony Formation and Invasion of CRPC Cells In Vitro 

While AURKA is a well-established oncogene, SPOP can act as a tumor-promoter or tumor-suppressor depending on the cell type. Nonetheless, multiple studies have shown that SPOP acts as a tumor suppressor in PCa by degrading its oncogenic substrates [21]. 

**Figure 5:**
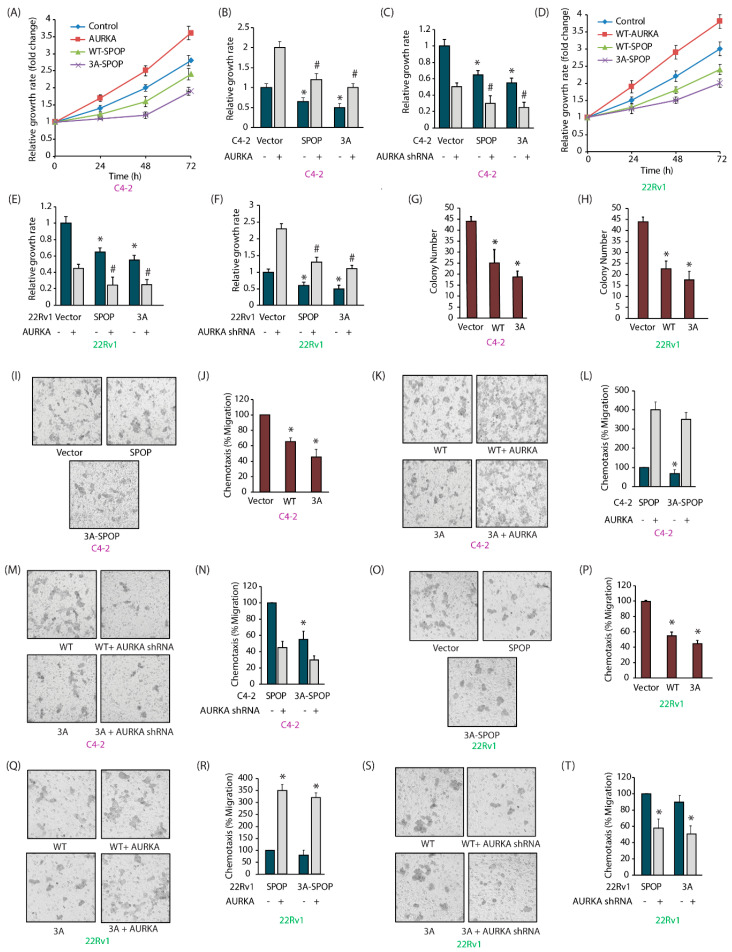
AURKA-mediated phosphorylation of SPOP promotes oncogenic phenotypes. (**A**) SPOP suppresses the proliferation of C4-2 cells. The proliferation of C4-2, AURKA-C4-2, SPOP-C4-2 and 3A-SPOP-C4-2 cells at 24 h, 48 h and 72 h was evaluated by MTT assay. (**B**) Impact of AURKA overexpression on the cell proliferation in C4-2, SPOP-C4-2 and 3A-SPOP-C4-2 cells. * and # indicate statistically significant differences compared to respective controls; *p* < 0.05. (**C**) AURKA knockdown diminished cell growth in C4-2, SPOP-C4-2 and 3A-SPOP-C4-2 cells. * and # indicate statistically significant difference compared to respective controls; *p* < 0.05. (**D**) Decrease in cell proliferation with SPOP overexpression in 22Rv1 cells. 22Rv1, AURKA-22Rv1, SPOP-22Rv1 and 3A-SPOP-22Rv1 cells were cultured for 24 h, 48 h, and 72 h and subjected to MTT assay. (**E**) AURKA overexpression enhances cell proliferation in 22Rv1, SPOP-22Rv1 and 3A-SPOP-22Rv1 cells. * and # indicate statistically significant difference compared to respective controls; *p* < 0.05. (**F**) AURKA knockdown decreases cell growth in 22Rv1, SPOP-22Rv1 and 3A-SPOP-22Rv1 cells. * and # indicate statistically significant difference compared to respective controls; *p* < 0.05. (**G**) Decrease in colony number with WT and 3A-SPOP overexpression as seen in C4-2 and (**H**) 22Rv1 cells. * *p* < 0.05 compared to vector-expressing control. (**I**) WT and 3A-SPOP inhibits cell migration in C4-2 cells. Representative images of chemotaxis assay. (**J**) Chemotaxis assay was performed in C4-2, SPOP-C4-2 and 3A-SPOP-C4-2 cells using Boyden chambers. Histogram shows mean ± SEM values from three independent experiments. * *p* < 0.05 compared to vector control. (**K**,**L**) AURKA overexpression increases cell motility in both SPOP-C4-2 and 3A-SPOP-C4-2 cells, although to a higher extent in the former as compared to the latter. (**M**,**N**) AURKA depletion inhibits cell motility in both SPOP-C4-2 and 3A-SPOP-C4-2 cells with a greater decrease in the latter. (**O**) Representative images and (**P**) quantitative data of chemotaxis assays measuring migration in 22Rv1, WT-SPOP-22Rv1 and 3A-SPOP-22Rv1 cells. (**Q**,**R**) AURKA overexpression increases cell motility in both SPOP-22Rv1 and 3A-SPOP-22Rv1 cells. (**S**,**T**) AURKA depletion inhibits cell motility in both SPOP-22Rv1 and 3A-SPOP-22Rv1 cells.

Therefore, we evaluated the consequences of AURKA-SPOP cross talk in endowing aggressive phenotypes in PCa cells. Ectopic expression of AURKA increased cellular proliferation in C4-2 cells (Figure 5A). In contrast, expression of 3A-SPOP was most effective in decreasing cell proliferation. When AURKA was overexpressed, C4-2 cells showed a much more robust response, compared to SPOP-C4-2 and 3A-SPOP-C4-2 cells, indicating that SPOP expression attenuates AURKA’s oncogenicity (Figure 5B). These results were confirmed by depletion of AURKA, which showed more significant impact on SPOP-C4-2 and 3A-SPOP-C4-2 cells, compared to parental C4-2 cells (Figure 5C). Comparable results were obtained in 22Rv1 cells (Figure 5D–F). 

The effect of SPOP phosphorylation was also observed in clonogenic assay. SPOP expression significantly decreased colony formation in C4-2 and 22Rv1 cells as compared to control cells (Figure 5G,H). 3A-SPOP cells showed more drastic inhibition as compared to WT-SPOP cells indicating that AURKA-mediated phosphorylation of SPOP promotes anchorage-independent growth of CRPC cells. 

### 2.8. AURKA-Mediated SPOP Phosphorylation Prevents Cell Migration

We explored the potential role of SPOP in cellular migration. As expected, SPOP overexpression decreased cell motility, which was more pronounced in 3A-SPOP overexpressing cells (Figure 5I,J). Furthermore, AURKA overexpression enhanced, and its knockdown decreased, cell migration in both SPOP-C4-2 and 3A-C4-2 cells (Figure 5K–N), but the effect was more significant in 3A-cells. Comparable results were obtained in 22Rv1 cells (Figure 5O–T). These results corroborate that AURKA-mediated phosphorylation of SPOP inhibits cell motility in CRPC cells. 

### 2.9. AURKA-Mediated SPOP Degradation Promotes c-Myc and AR Stability

**Figure 6:**
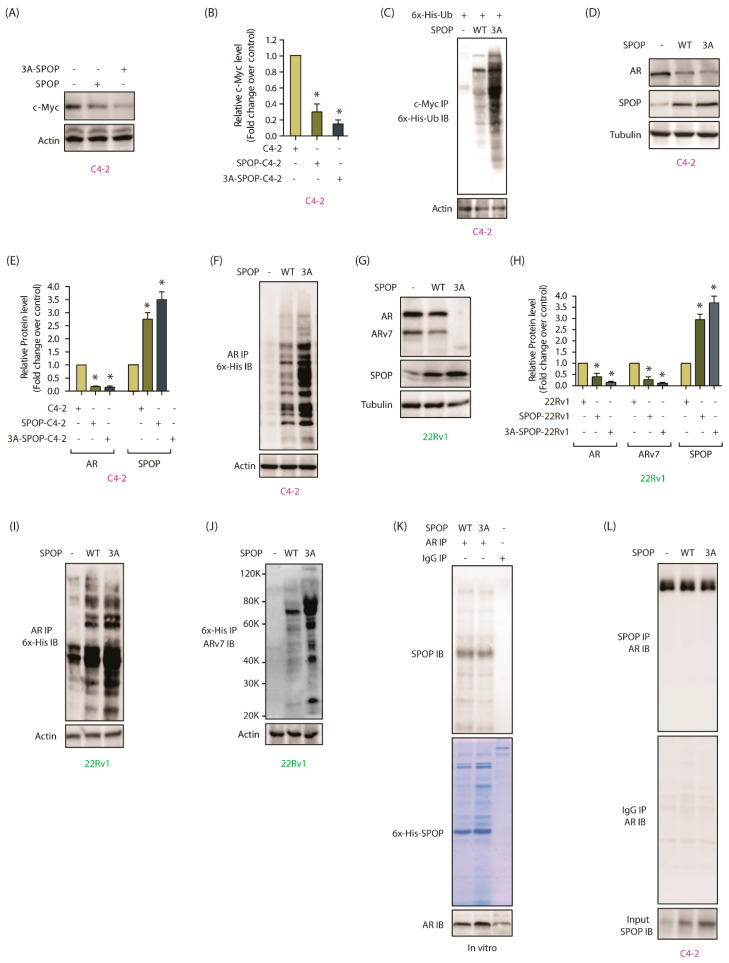
AURKA promotes AR, ARv7 and c-Myc stability by degrading SPOP. (**A**) WT-SPOP and 3A-SPOP decrease c-Myc protein expression levels. (**B**) Histogram shows changes in c-Myc protein levels. The data presented as mean ± SEM obtained from three independent experiments. * *p* < 0.05 vs. C4-2 control cells. (**C**) 3A-SPOP promotes higher ubiquitylation of c-Myc protein as compared to WT-SPOP. (**D**) Both WT-SPOP and 3A-SPOP decreases AR protein levels in C4-2 cells. (**E**) Histogram shows change in AR and SPOP protein levels. The data are presented as mean ± SEM obtained from three independent experiments. * *p* < 0.05 vs. C4-2 control cells. (**F**) 3A-SPOP promotes more robust ubiquitylation of AR protein, compared to WT-SPOP in C4-2 cells. (**G**) Ectopic expression of 3A-SPOP decreases AR and ARv7 protein levels more than WT-SPOP in 22Rv1 cells. (**H**) Histogram shows changes in AR and ARv7 protein levels in 22Rv1, SPOP-22Rv1 and 3A-SPOP-22Rv1 cells. The data are presented as mean ± SEM obtained from three independent experiments. * *p* < 0.05 vs. 22Rv1 control cells. (**I**) 3A-SPOP promotes more robust ubiquitylation of AR in 22Rv1 cells compared to WT-SPOP. (**J**) 3A-SPOP promotes more robust ubiquitylation of ARv7 in 22Rv1 cells compared to WT-SPOP. (**K**) WT and 3A-SPOP bind AR equally well. In vitro pull-down assay using recombinant SPOP and AR proteins were used to check AR and SPOP association. AR on beads was incubated with 6x-His-SPOP (WT and 3A) and binding analyzed. (**L**) SPOP and AR binding in C4-2, SPOP and 3A-SPOP-expressing C4-2 cells. SPOP protein was immunoprecipitated from the above-mentioned cells and AR binding analyzed by immunoblotting (top panel). IgG IP followed by AR IB is shown in the same line in the middle panel. Lower panel shows SPOP levels as input.

We next determined the impact of SPOP phosphorylation by AURKA at the molecular level by focusing on three highly oncogenic targets of SPOP- c-Myc, AR and ARv7. As mentioned before, AR is the central player in CRPC pathogenesis and c-Myc positively regulates AR transcription and protein stability [27]. In addition, c-Myc regulates protein stability of AR splice variants further exacerbating the disease. Ectopically expressed 3A-SPOP in C4-2 cells showed robust downregulation of c-Myc, as compared to wild-type SPOP, highlighting the significance of phosphorylation-mediated downregulation of SPOP (Figure 6A,B and Figure S6A). As SPOP ubiquitylates its substrates, we investigated the ubiquitylation of c-Myc in C4-2, SPOP-C4-2 and 3A-SPOP-C4-2 cells, which revealed substantially higher ubiquitylation of c-Myc in phospho-resistant SPOP expressing cells, compared to SPOP-C4-2 cells (Figure 6C). These results underscore that SPOP downregulation by AURKA is a vital step in c-Myc augmentation. AR levels were examined in C4-2 cells with or without wild-type and 3A-SPOP expression. Both wild-type and 3A-SPOP decreased AR to a minimal level. As C4-2 cells show low AR expression, it could account the lack of noticeable difference in AR levels between SPOP-C4-2 and 3A-SPOP-C4-2 cells (Figure 6D,E and Figure S6D). However, when we examined the ubiquitylation of AR in these cells, 3A-SPOP-expressing cells showed much stronger ubiquitylation (Figure 6F), confirming that AURKA inhibits AR degradation via SPOP depletion. We also investigated the consequences of wild-type and mutant SPOP expression in 22Rv1 cells, which showed loss of AR expression as compared to C4-2 cells. While wild-type SPOP substantially decreased AR levels, 3A-SPOP completely obliterated it (Figure 6G,H and Figure S6G). Consistent with this result, 3A-SPOP induced higher ubiquitylation of AR, compared to wild-type SPOP (Figure 6I). 

AR splice variant, ARv7, lacks SPOP binding motif and thus is not a direct substrate of SPOP. Nevertheless, ARv7 levels were drastically reduced by the expression of WT-SPOP and 3A-SPOP in 22Rv1 cells (Figure 6G,H). Furthermore, 3A-SPOP caused robust ubiquitylation of ARv7 further supporting that AURKA-mediated SPOP phosphorylation controls ARv7 levels as well in cells (Figure 6J).

SPOP binds its substrates via the MATH domain. As all three AURKA sites are within this domain (Figure 4C), we explored whether phospho-resistant mutations influence its binding with AR both in vitro and in cells. AR immune complex was isolated from cells and incubated with either 6x-His-tagged wild-type or mutant SPOP, both of which showed similar binding to AR (Figure 6K). These results were confirmed in cells, where SPOP IP from all three cell-types (C4-2, WT-SPOP-C4-2 and 3A-SPOP-C4-2) showed equivalent AR binding (Figure 6L). These results validate that AURKA downregulates AR by degrading SPOP and not by attenuating its binding to AR.

### 2.10. AURKA-SPOP Feedback Loop in Androgen-Sensitive LNCaP Cells

**Figure 7:**
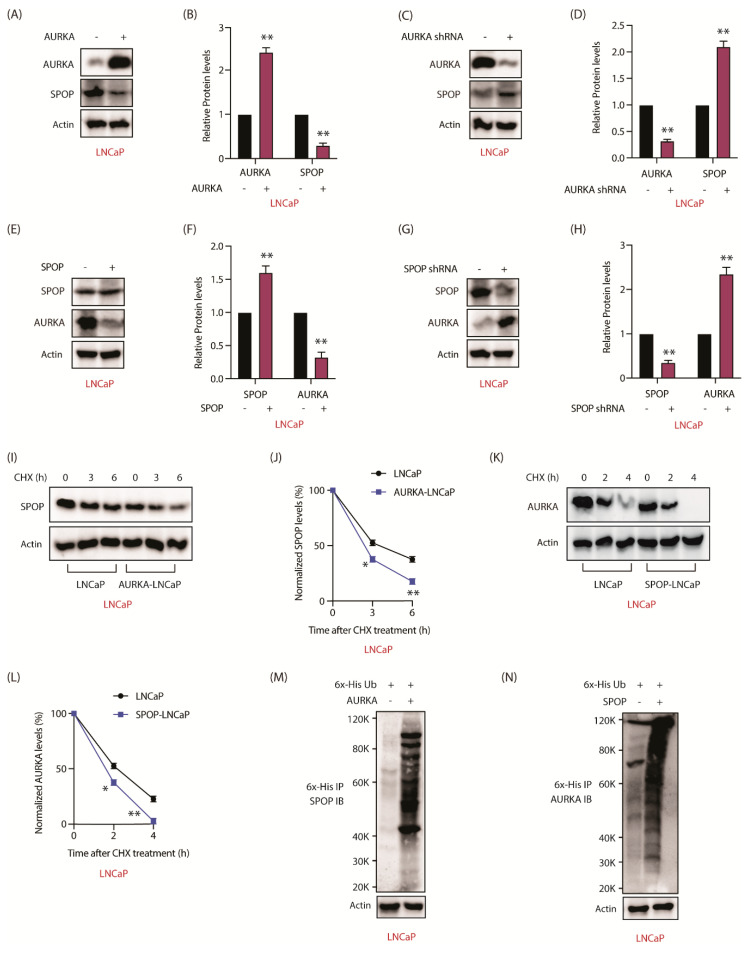
AURKA and SPOP are involved in a negative feedback loop in LNCaP cells as well. (**A**) AURKA negatively regulates SPOP in LNCaP cells. (**B**) Histogram showing change in SPOP levels with AURKA overexpression. The data are presented as mean ± SEM obtained from three independent experiments. ** *p* < 0.01 vs. LNCaP control cells. (**C**) AURKA silencing increases SPOP in LNCaP cells. LNCaP cells were infected with either scrambled shRNA or shRNA targeting AURKA lentivirus, followed by IB. (**D**) Histogram shows change in SPOP levels with AURKA knockdown. The data are presented as mean ± SEM obtained from three independent experiments. ** *p* <0.01 vs. LNCaP control cells. (**E**) Overexpression of SPOP decreased the levels of AURKA in LNCaP cells. (**F**) Histogram shows change in AURKA levels with SPOP overexpression. The data are presented as mean ± SEM obtained from three independent experiments. ** *p* < 0.01 vs. LNCaP control cells. (**G**) SPOP silencing increases AURKA levels in LNCaP cells. (**H**) Histogram shows change in AURKA levels with SPOP knockdown. The data are presented as mean ± SEM obtained from three independent experiments. ** *p* < 0.01 vs. LNCaP control cells. (**I**) AURKA increases SPOP degradation in LNCaP cells. LNCaP and AURKA-LNCaP cells were treated with cycloheximide (10 μM) for 3 and 6 h and SPOP levels analyzed. (**J**) Graphical representation of SPOP degradation rate in LNCaP cells. The data are presented as mean ± SEM obtained from three independent experiments. * *p* < 0.05, ** *p* <0.01 vs. LNCaP control cells. (**K**) SPOP increases AURKA degradation in LNCaP cells. (**L**) Graphical representation of AURKA degradation rate in LNCaP cells. The data are presented as mean ± SEM obtained from three independent experiments. * *p* < 0.05, ** *p* < 0.01 vs. LNCaP control cells. (**M**) AURKA degrades SPOP by enhancing its ubiquitylation. LNCaP cells were co-infected with 6x-His-ubiquitin and AURKA retrovirus for 30 h followed by MG132 treatment for 12 h. 6x-His-tagged ubiquitylated proteins were isolated and ubiquitylation analyzed using SPOP antibody. (**N**) SPOP degrades AURKA via increased ubiquitylation. LNCaP cells were co-infected with 6x-His-ubiquitin and SPOP retrovirus for 30 h followed by MG132 treatment for 12 h. 6x-His-tagged ubiquitylated proteins were isolated and ubiquitylation analyzed using AURKA antibody. Each experiment was done at least three independent times and representative data are shown.

We next explored whether AURKA regulates SPOP in androgen-sensitive LNCaP cells. When AURKA was overexpressed, SPOP levels decreased in LNCaP cells (Figure 7A,B and Figure S7A). Similarly, when AURKA was depleted, SPOP levels increased (Figure 7C,D and Figure S7C), indicating that AURKA negatively regulates SPOP in androgen-sensitive cells as well. Furthermore, SPOP also negatively regulates AURKA, as SPOP overexpression decreased, and its depletion increased AURKA levels (Figure 7E–H and Figure S7E,G). 

To investigate whether SPOP regulation was due to increased degradation, AURKA was overexpressed in LNCaP cells, which decreased the stability of SPOP (Figure 7I,J and Figure S7I). Similarly, SPOP overexpression decreased the stability of AURKA in LNCaP cells (Figure 7K,L and Figure S7K). Finally, we observed that AURKA increases the ubiquitylation of SPOP and vice versa (Figure 7M,N). Together, these results show that AURKA-SPOP feedback loop occurs both in androgen-sensitive and CRPC cells.

### 2.11. AURKA-Mediated SPOP Phosphorylation Correlates with Tumor Progression and EMT In Vivo

**Figure 8:**
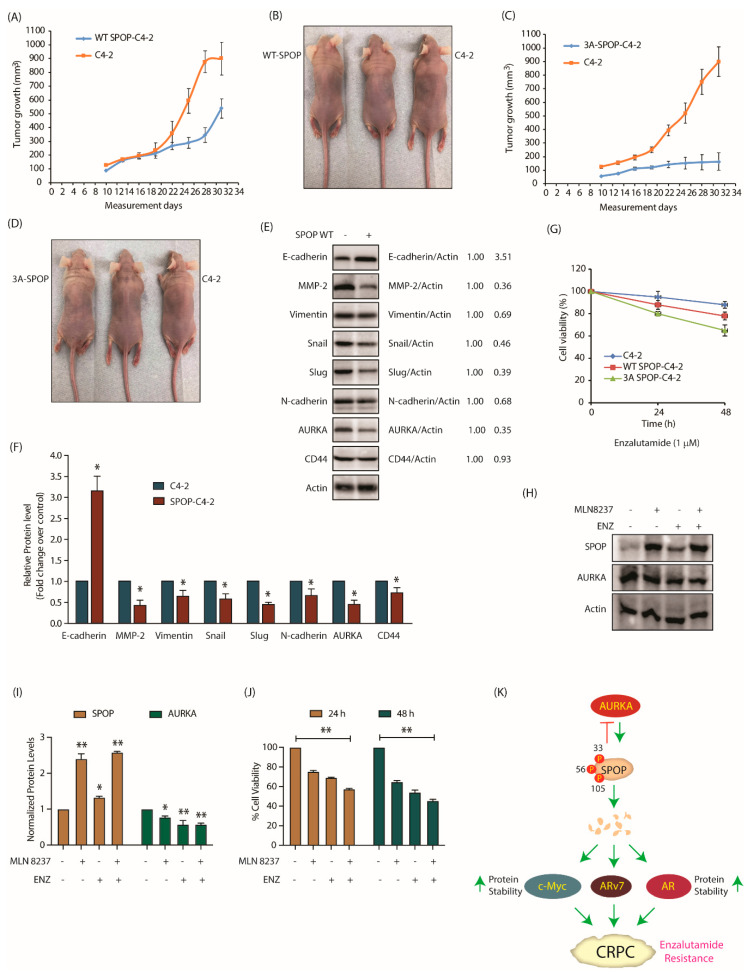
SPOP degradation by AURKA promotes tumorigenesis, EMT and enzalutamide-resistance. (**A**) SPOP overexpression prevents tumor growth in vivo. Growth curves of tumor in nude mice inoculated with C4-2 and SPOP-C4-2 cells on right and left shoulders, respectively. The mean value ± SEM values were from three animals in each group. (**B**) Representative images of tumor bearing nude mice. Pictures were taken 32 days after injection. (**C**) Growth curves of tumor obtained from nude mouse injected with C4-2 and 3A-SPOP-C4-2 cells. (**D**) Representative image showing mouse with tumor. The pictures were taken 32 days following inoculation. (**E**) Expression of EMT markers in tumor tissues obtained from C4-2 and SPOP-C4-2 cells injected in mice. (**F**) Histogram shows expression levels of EMT markers in tumor isolated from C4-2 and SPOP-C4-2 injected mice. The data are presented as mean ± SEM values collected from three independent experiments. * *p* < 0.05 was analyzed using two-way ANOVA. (**G**) 3A-SPOP-expressing cells are more sensitive to enzalutamide (1 μM, treated for 48 h), compared to WT-SPOP-C4-2 cells. (**H**) Changes in SPOP levels with treatments of MLN8237 (1 μM, treated for 12 h), and enzalutamide (1 μM, treated for 12 h). (**I**) Quantification of protein levels as a function of drug treatment obtained from three independent experiments, * *p* < 0.05, ** *p* < 0.01. (**J**) Loss of cell viability with treatments of MLN8237 (1 μM, treated for 24 and 48 h), and enzalutamide (1 μM, treated for 24 and 48 h). Data obtained from three independent experiments, ** *p* < 0.01. (**K**) Schematic model depicting the consequences of AURKA-SPOP signaling in CRPC pathogenesis.

To examine the anti-tumorigenic potential of SPOP in vivo, we injected C4-2 and SPOP-C4-2 cells into nude mice and monitored xenograft tumor growth. SPOP overexpression in C4-2 cells resulted in reduced tumor growth as compared to C4-2 cells (Figure 8A,B). Simultaneously, in another set of experiments, nude mice were injected with C4-2 and 3A-SPOP-C4-2 cells on right and left shoulders, respectively. C4-2 cells formed robust tumors as before, whereas 3A-SPOP-C4-2 cells showed absolutely no tumor formation (Figure 8C,D). Thus, phospho-resistant SPOP has higher tumor-suppressing potential as compared to wild type SPOP, presumably due to its resistance to AURKA-mediated degradation.

As AURKA promotes EMT, we next assessed the contribution of SPOP in this process. C4-2 and WT-SPOP tumors were isolated, however, as 3A-SPOP-expressing cells did not form tumors, they could not be analyzed. Various EMT inducing proteins such as MMP-2, vimentin, snail, slug, N-cadherin and CD44 were analyzed in C4-2 and WT-SPOP tumor lysate. All these proteins showed reduced expression in SPOP-expressing xenografts, compared to C4-2 xenografts. In addition, we also analyzed E-cadherin, an epithelial marker, which showed much higher levels in SPOP-overexpressing xenografts. These results confirm that SPOP inhibits EMT in vivo. Importantly, AURKA expression was significantly reduced in SPOP-expressing xenografts, confirming the negative feedback loop in vivo (Figure 8E,F and Figure S8E).

Finally, we investigated whether SPOP phosphorylation by AURKA affects enzalutamide resistance. 3A-SPOP-expressing cells were most sensitive to enzalutamide as compared to C4-2 and SPOP-C4-2 cells, implying that SPOP degradation via phosphorylation is also an important determinant in promoting drug resistance (Figure 8G).

To confirm this finding, we treated C4-2 cells with MLN8237 and enzalutamide independently and in combination. AURKA inhibition reinstated the stability of wild-type SPOP as expected (Figure 8H,I and Figure S8I). Notably, enzalutamide treatment also increased SPOP levels. Furthermore, combination of MLN8237 and enzalutamide showed enhancement in cytotoxicity (Figure 8J). These results indicate that SPOP upregulation is one of the mechanisms by which enzalutamide exerts its efficacy. 

## 3. Discussion

SPOP is an adaptor protein for RBX1-CUL3 E3 ubiquitin ligase complex. SPOP contains an N-terminal MATH domain (residues 28–166) which recruits substrates, followed by a BTB domain (residues 190–297) which mediates oligomerization and binds Cul3, an E3 ubiquitin ligase (Figure 4C) [26]. SPOP mutations occur early in PCa pathogenesis. Most of the known SPOP mutations in PCa are at the surface of the MATH domain, which diminishes its ability to bind its targets, thereby promoting invasion, proliferation, and immune escape in vivo [28]. SPOP binds to the hinge region of AR expediting its degradation [22]. Interestingly, ARv7 lacks the hinge region, but SPOP can still degrade it in the presence of full-length AR, presumably by forming heterodimers with AR [29]. Furthermore, SPOP can indirectly inhibit AR transcriptional output by degrading steroid receptor coactivator 3 (SRC-3) [30]. Thus, both SPOP ablation and SPOP mutants expressing xenografts display higher AR levels in vivo [29]. Cancer Genome Atlas (TCGA) analysis using 333 primary prostate carcinomas revealed that mutant SPOP-bearing tumors have the highest levels of AR-induced transcripts, indicating that upregulation of AR pathway is the most significant outcome of SPOP inactivation in PCa [31]. Not surprisingly, several clinical studies have shown that patients with prostate tumors bearing SPOP mutations are highly sensitive and show better response to androgen signaling inhibitors therapy, nevertheless it does not translate to survival benefit [32,33]. 

Importantly, a vast majority of prostate tumors that lack SPOP mutations still show reduced SPOP levels, implying that SPOP downregulation is essential for PCa progression [24]. While numerous downstream substrates of SPOP have been identified, there is no molecular mechanism or regulator known for wild-type SPOP protein in any cancer. This study uncovered the first post-translational regulation of SPOP, which is triggered by AURKA. As AURKA is highly expressed in a vast majority of PCa, we postulate that AURKA plays a critical role in degrading SPOP, thereby contributing to disease progression. 

SPOP was identified as the direct target of AURKA using a chemical-genetic strategy. This method entails an analog-sensitive space-creating mutation in the active site of the kinase of interest, coupled with an orthogonal ATP analog, which bears a bulky group at the N-6-position [34,35,36,37]. We had generated analog-sensitive AURKA (aka AURKA-as7) in our previous study [20]. AURKA-as7 preferentially uses N-6-Phenthyl ATP. Using this approach, we have previously identified many substrates of AURKA [11,12,13,14,20]. Recently, we reported that AURKA directly phosphorylates multifunctional protein YBX1, which stabilizes it and promotes its nuclear translocation. In turn, YBX1 also stabilizes AURKA. The feedback loop between YBX1 and AURKA contributes significantly to EMT, drug-resistance and tumorigenesis in vivo [14]. 

AURKA directly binds and phosphorylates SPOP at three different positions—S33, T56 and S105 —all of which are within the MATH domain, although none of these sites are known to be mutated. Instead, this study revealed that post-translational modification of the MATH domain is yet another key mechanism that downregulates SPOP, promoting disease progression. Phospho-resistant SPOP possesses enhanced stability compared to wild-type rendering it highly effective in suppressing oncogenic phenotypes, EMT and enzalutamide resistance in CRPC cells (Figure 8K). In vivo, it fully abrogates tumorigenesis, thereby underscoring the relevance of AURKA-mediated regulation of SPOP in CRPC pathogenesis. This is the first study where SPOP has been shown to be post-translationally modified by any kinase.

This study has provided new molecular links between AURKA and AR. We have shown that AURKA upregulates c-Myc via SPOP, which in turn is known to increase AR transcription. Additionally, AURKA regulates AR and ARv7 stability via SPOP. Surprisingly, this study also uncovered AURKA as a novel substrate of SPOP. SPOP-AURKA interaction allows SPOP to control AURKA stability and reduce its oncogenic potential in PCa. SPOP targets AURKA for ubiquitylation and proteasomal degradation thus preventing cell growth, and the migratory and invasion abilities of CRPC cells. Finally, we show that SPOP is upregulated upon enzalutamide treatment. Not surprisingly, phospho-resistant SPOP cells displayed higher sensitivity to enzalutamide compared to control cells. Thus, we postulate AURKA inhibition provides a powerful tool to retain WT-SPOP in a vast majority of CRPC patients using AURKA inhibitors ± enzalutamide, thereby treating and inhibiting disease progression. 

## 4. Materials and Methods

### 4.1. Cell Lines and Cell Culture

PCa cell lines (22Rv1, C4-2, LNCaP), HEK-293T and Phoenix cell lines were acquired from American Typical Culture Center (ATCC; Manassas, VA, USA). LNCaP, 22Rv1 and C4-2 cells were maintained in RPMI-1640 media while the other two cell lines were maintained in DMEM medium supplemented with 10% FBS, 1 × penicillin-streptomycin. All the cells were grown at 37 °C with 5% CO_2_ in a humidified incubator. Details of antibodies are provided in Table S1.

### 4.2. shRNA Construction and Lentivirus Infection

AURKA shRNA was cloned in our previous study [20]. SPOP shRNA was designed and cloned in pLKO vector [38]. The primers are included in Supplementary Table S2. Lentivirus were produced as before [39]. 

### 4.3. Plasmid Cloning and Purification 

SPOP was cloned into the TAT-HA vector at the BamHI and XhoI sites. SPOP mutants were generated using site-directed mutagenesis. 6x-His-tagged SPOP was expressed in BL21 *E. coli* competent cells, purified using Ni-NTA beads and were confirmed using SPOP monoclonal antibody (Santa Cruz Biotechnology, Dallas, TX, USA). AURKA was cloned, expressed and purified from SF9 insect cells [20]. AURKA and SPOP retrovirus were generated to infect PCa cells using method reported previously [40].

### 4.4. AURKA Kinase Assays

AURKA kinase assay was assayed with AURKA and TPX2 purified using Ni-NTA beads. The kinase was activated by incubating in kinase assay buffer (50 mM Tris, 10 mM MgCl_2_) with 100 μM of ATP for 2 h at 30 ℃. Thereafter, the beads were washed with kinase assay buffer to remove extra ATP. For phosphorylation experiments, 2 μg of 6x-His-tagged recombinant protein (wild-type or mutant SPOP) was mixed with AURKA-TPX2 in the presence of 1 μCi of [γ-^32^P] ATP for 30 min. The reactions were terminated with SDS loading dye, boiled and resolved by SDS-PAGE and finally exposed for autoradiography.

### 4.5. Immunofluorescence

Immunofluorescence was performed as described before [41]. Cells were captured using Nikon Eclipse E600 microscope (Nikon Instruments, Melville, NY, USA).

### 4.6. RNA Extraction and qPCR

Total RNA was isolated using Trizol reagent from control and shRNA treated cells following the instructions of the manufacturer. RNA was transcribed using the RT-PCR kit (Promega, Madison, WI, USA). Gene expression was detected by quantitative real-time RT-PCR (qRT-PCR) using 2 × SYBR Green master mix (BioRad, Hercules, CA, USA). The primer sequences are presented in Supplementary Table S3. Each qPCR experiment was carried out three independent times in triplicate.

### 4.7. In Vitro Ubiquitylation Assay

For ubiquitylation experiments, both 6x-His-Ubiquitin and AURKA or SPOP retrovirus were co-infected in PCa cells for 30 h. Subsequently, 10 μM MG132 was added for an additional 12 h to stabilize ubiquitylated proteins. Cells were lysed and the cell lysate was incubated with pre-washed Ni-NTA beads or specific antibodies (Table S1) for 4 h to pull-down 6x-His-ubiquitin-conjugated proteins. Beads were washed followed by SDS-PAGE and immunoblot analysis to detect ubiquitylated proteins.

### 4.8. Chemotaxis Assay

Cell migration was performed using Boyden chambers as reported previously [42]. The assay was independently repeated at least three times including technical replicates.

### 4.9. Cell Proliferation Assay

The MTT assay was conducted as reported in our previous publication [13]. In brief, PCa cells at a seeding density of 2000 cells/100 µL/well was grown in a 96-well plate. 20 µL MTT solution (5 mg/mL) was added to each well after indicated time periods (24, 48 and 72 h), and further incubated for 4 h at 37 °C. After incubation the culture medium was removed and 200 μL DMSO was added to each well and absorbance was read at 570 nm using an ELISA plate reader.

### 4.10. Clonogenic Assay

Clonogenic assay was conducted as described earlier [43]. RPMI containing 0.5% agarose gel and 10% FBS was added to a 6-well plate and incubated at RT for 1 h. PCa cells (5 × 10^3^) were re-suspended in RPMI medium containing 0.3% agarose gel and 10% FBS and seeded on the top of base agar. Top agar was covered with 500 µL of RPMI. The plates were then incubated at 37 °C for 3–4 week with fresh media added twice a week. Thereafter colonies washed with PBS, fixed and stained with crystal violet (0.1%). Colonies were observed and counted using a light phase-contrast microscope. 

### 4.11. Mouse Xenograft Model

All experiments were performed in accordance with protocols (protocol # 1111000292; approval date 01/07/2019) approved by the Purdue Animal Care and Use Committee (PACUC) of Purdue University. Animals were housed in a pathogen-free animal facility with 3 mice per cage. PCa cells (1 × 10^6^ cells per mouse) were mixed with an equal volume of Matrigel (Invitrogen, Carlsbad, CA, USA) and inoculated subcutaneously into right and left shoulders of nude mice (Taconic Biosciences, Cambridge City, IN, USA). After ten days, tumor volumes were measured every alternate day with digital Vernier calipers. Mice were euthanized on day 32 and tumor tissues were harvested and flash-frozen in liquid nitrogen for further studies.

### 4.12. Statistical Analysis

Data were presented as mean ± SEM of at least three or more experiments. Statistical analysis was determined by the one-way analysis of variance (ANOVA) followed by Bonferroni’s post hoc test using GraphPad Prism (version 6.07) software (GraphPad Software, San Diego, CA, USA). Statistical significance was considered at *p* < 0.05.

## 5. Conclusions

While prostate tumors bearing genomic mutations of SPOP can only be treated using gene therapy at present, identification of AURKA as an upstream regulator of SPOP provides a powerful therapeutic opportunity for large number of PCa patients which harbor wild-type SPOP by targeting AURKA using small molecule inhibitors. 

## Supplementary Materials

The following are available online at https://www.mdpi.com/2072-6694/12/11/3247/s1, Figure S1: Full length western blot images of Figure 1; Figure S2: Full length western blot images of Figure 2; Figure S3: Full length western blot images of Figure 3; Figure S4: Full length western blot images of Figure 4; Figure S5: Immunofluorescence images assessing WT and 3A-SPOP localization in C4-2 and 22Rv1 cells; Figure S6: Full length western blot images of Figure 6; Figure S7: Full length western blot images of Figure 7; Figure S8: Full length western blot images of Figure 8. Table S1: List of antibodies used in this study; Table S2: Sequence of SPOP shRNA; Table S3: Sequences of real time qPCR primers.
