# Supplementary material for: Molecular Interplay between AURKA and SPOP Dictates CRPC Pathogenesis via Androgen Receptor
Source: Cancers (Basel). 2020 Nov 4;12(11):3247. doi: 10.3390/cancers12113247 (PMC7693105; doi:10.3390/cancers12113247)
Supplement: Supplementary file 1 [file cancers-12-03247-s001.pdf]

cancers

Supplementary Materials

Molecular Interplay between AURKA and SPOP Dictates CRPC Pathogenesis via Androgen Receptor

Kumar Nikhil, Mohini Kamra, Asif Raza, Hanan S. Haymour and Kavita Shah

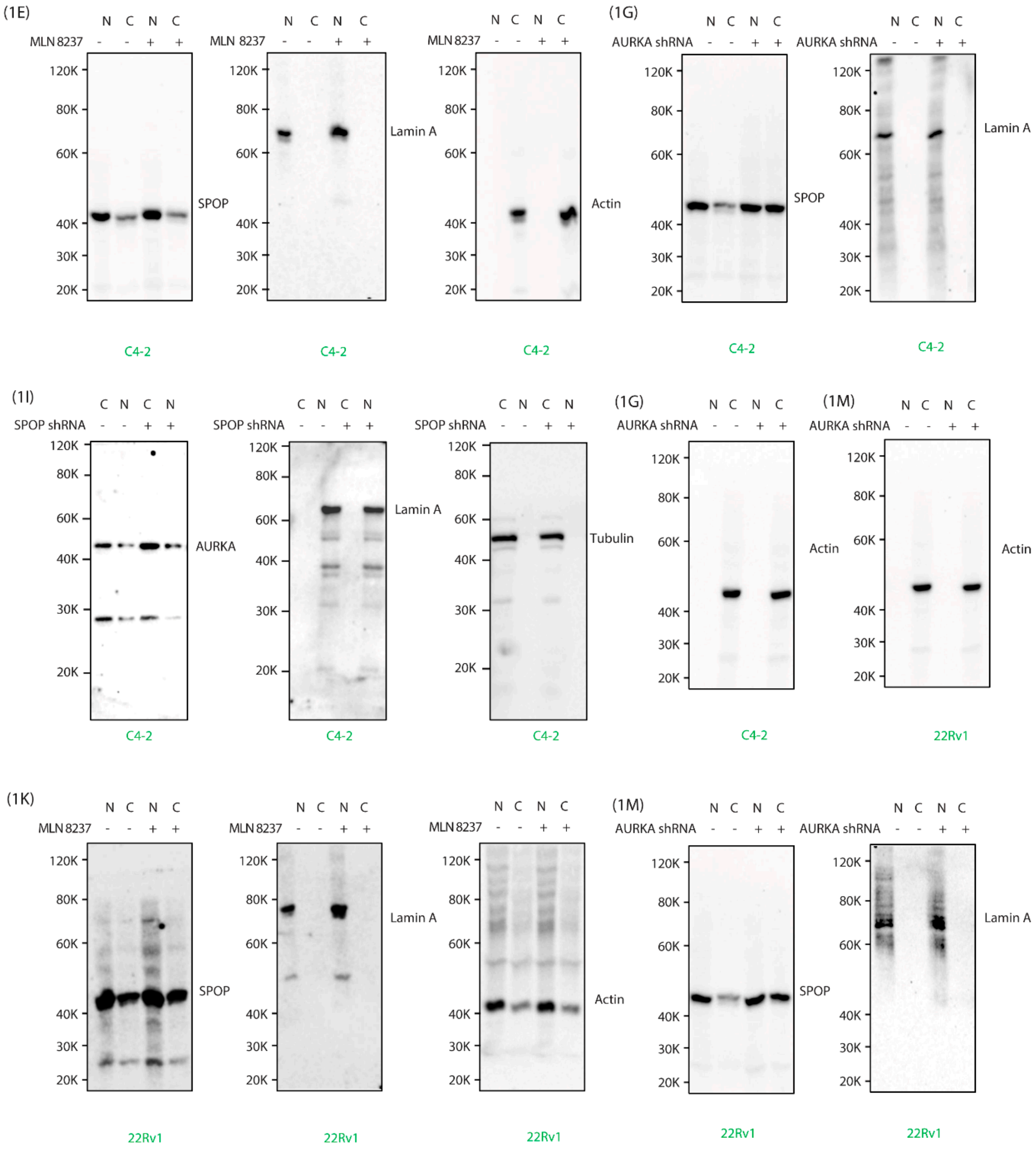

Figure S1. (1E) Full length western blot images of SPOP, Lamin A and Actin from fractionated lysates of vector and MLN8237-treated C4-2 cells. (1G) Full length western blot images of SPOP, Lamin A and Actin from fractionated lysates of scrambled and AURKA shRNA-treated C4-2 cells. (1I) Full length western blot images of AURKA, Lamin A and Tubulin from fractionated lysates of scrambled and SPOP shRNA-treated C4-2 cells. (1K) Full length western blot images of SPOP, Lamin A and

Actin from fractionated lysates of vector and MLN8237-treated 22Rv1 cells. (1M) Full length western blot images of SPOP, Lamin A and Actin from fractionated lysates of scrambled and AURKA shRNA-treated 22Rv1 cells.

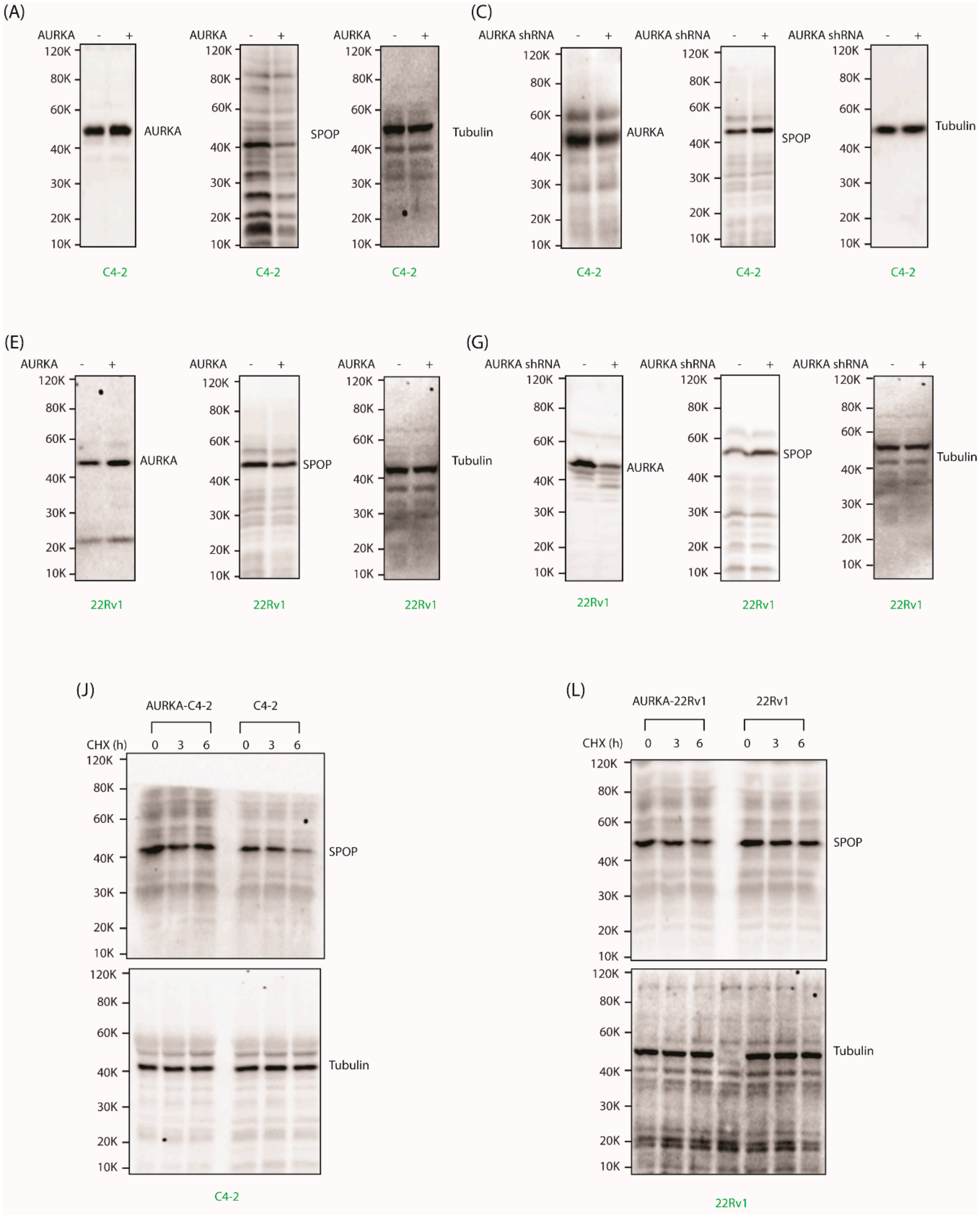

Figure S2. (2A) Full length western blot images of Figure 2A. (2C) Full length western blot images of Figure 2C. (2E) Full length western blot images of Figure 2E. (2G) Full length western blot images of Figure 2G. (2J) Full length western blot images of Figure 2J. (2L) Full length western blot images of Figure 2L.

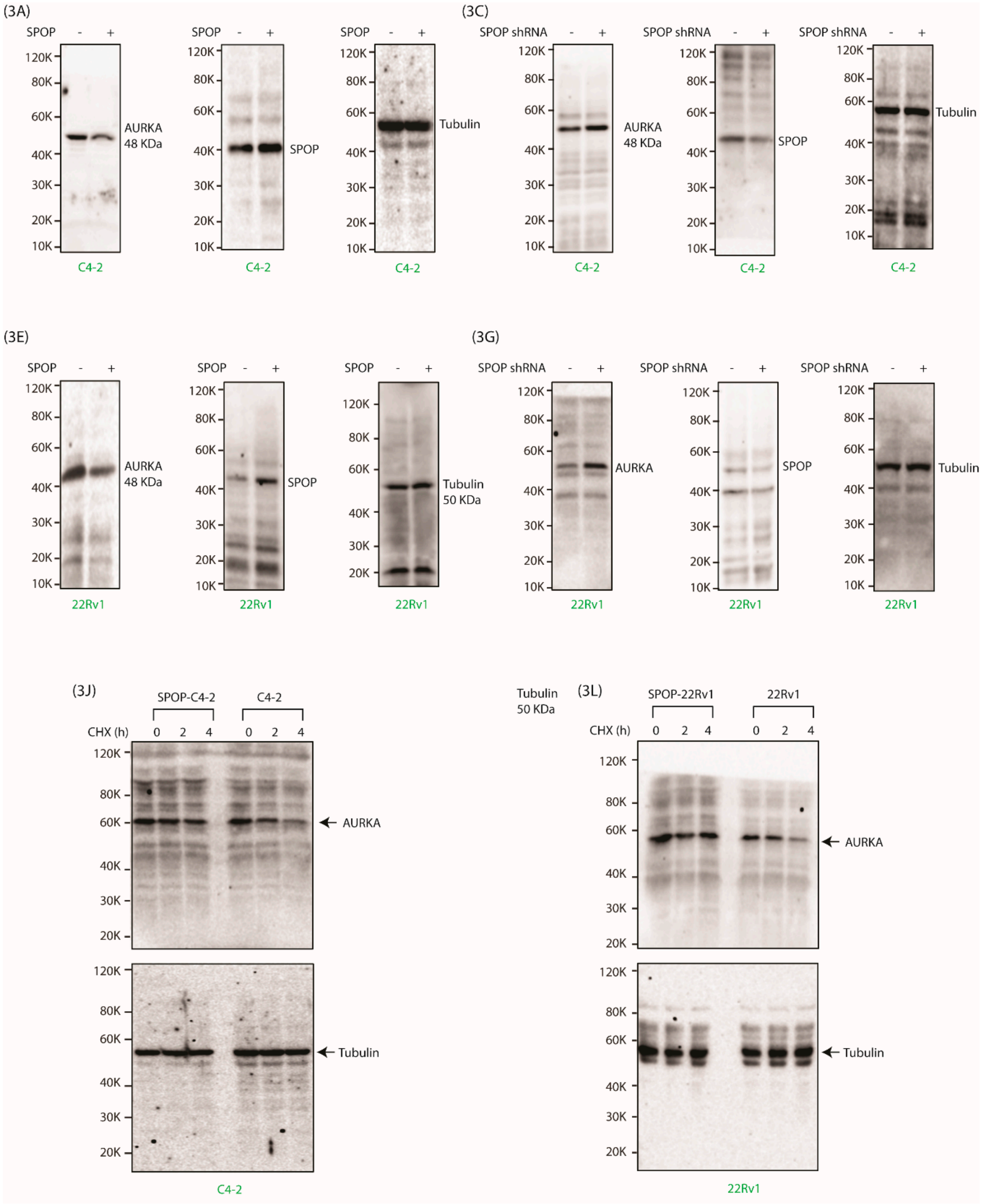

Figure S3. (3A) Full length western blot images of Figure 3A. (3C) Full length western blot images of Figure 3C. (3E) Full length western blot images of Figure 3E. (3G) Full length western blot images of Figure 3G. (3J) Full length western blot images of Figure 3J. (3L) Full length western blot images of Figure 3L.

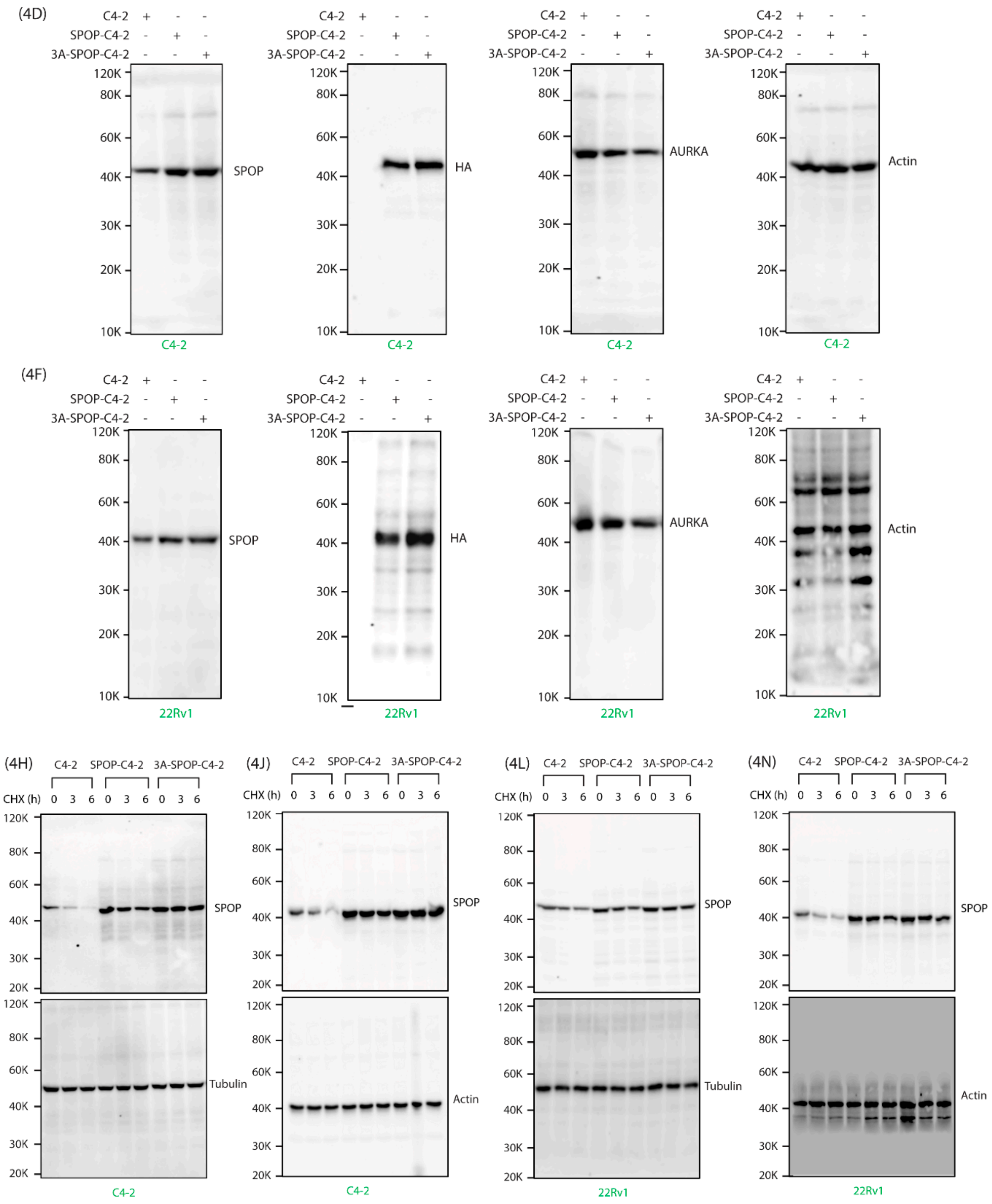

Figure S4. (4D) Full length western blot images of Figure 4D. (4F) Full length western blot images of Figure 4F. (4H) Full length western blot images of Figure 4H. (4J) Full length western blot images of Figure 4J. (4L) Full length western blot images of Figure 4L. (4N) Full length western blot images of Figure 4N.

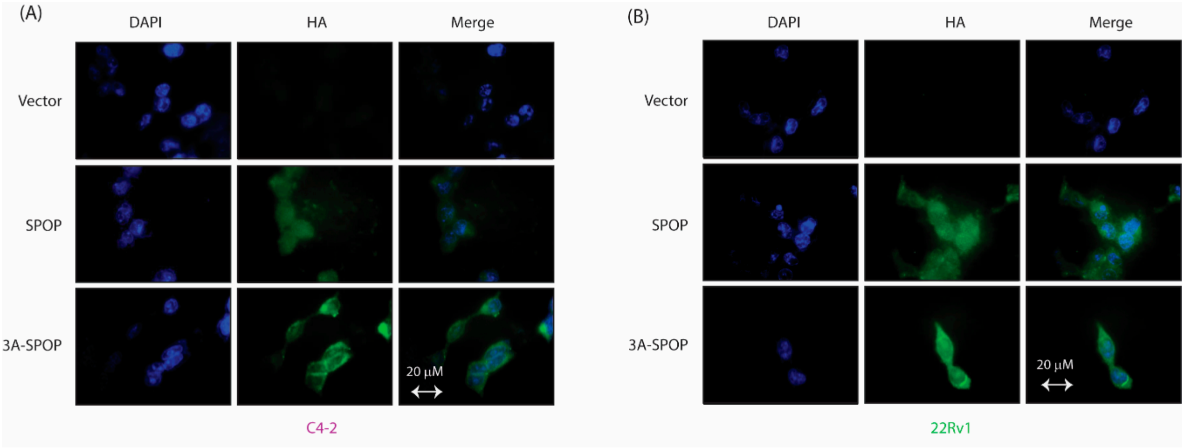

Figure S5. (5A) Immunofluorescence images assessing SPOP localization using HA expression in vector, SPOP-WT and SPOP 3A treated C4-2 cells. (5B) Immunofluorescence images assessing SPOP localization using HA expression in vector, SPOP-WT and SPOP 3A treated 22Rv1 cells.

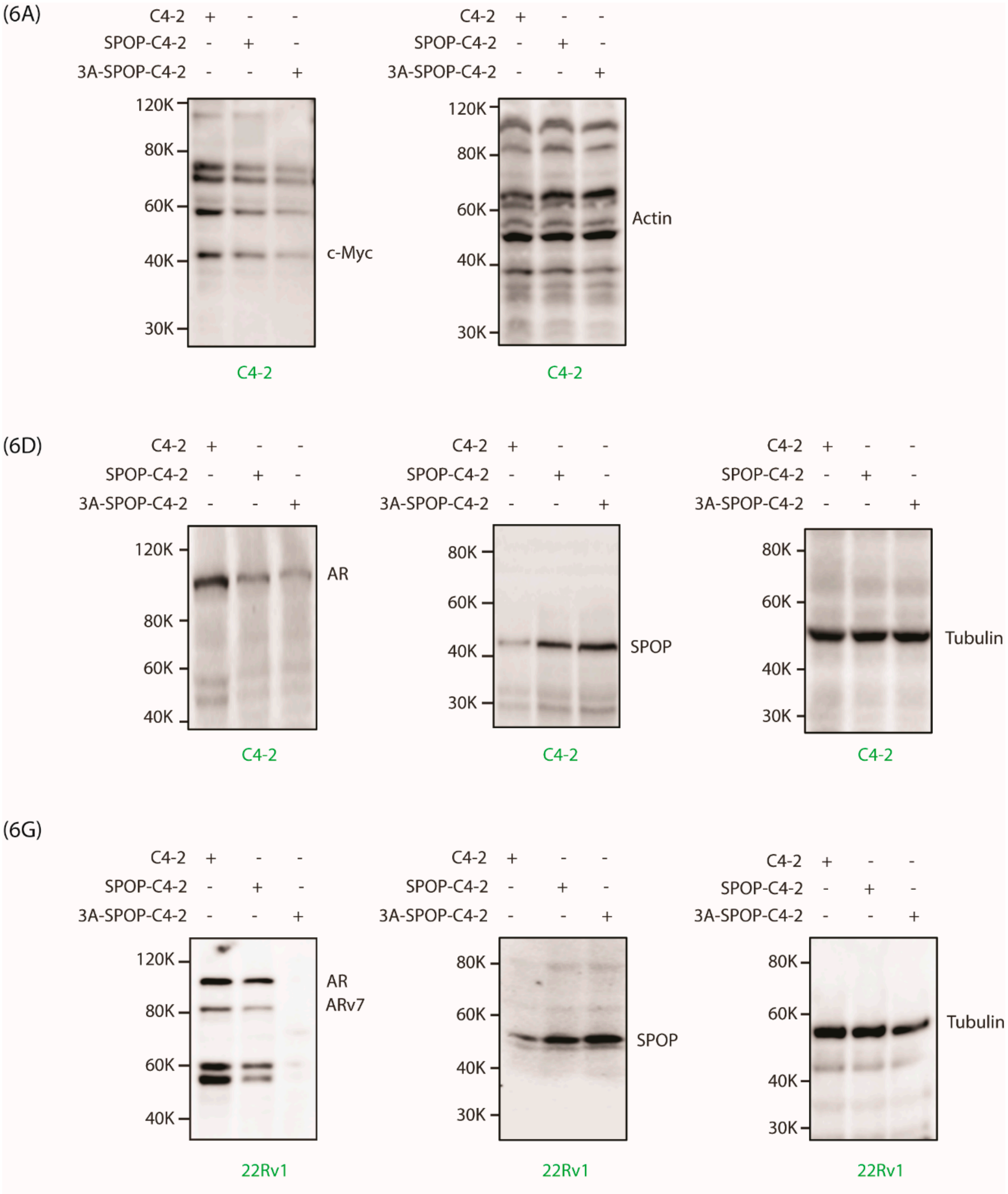

Figure S6. (6A) Full length western blot images of Figure 6A. (D) Full length western blot images of Figure 6D. (6G) Full length western blot images of Figure 6G.

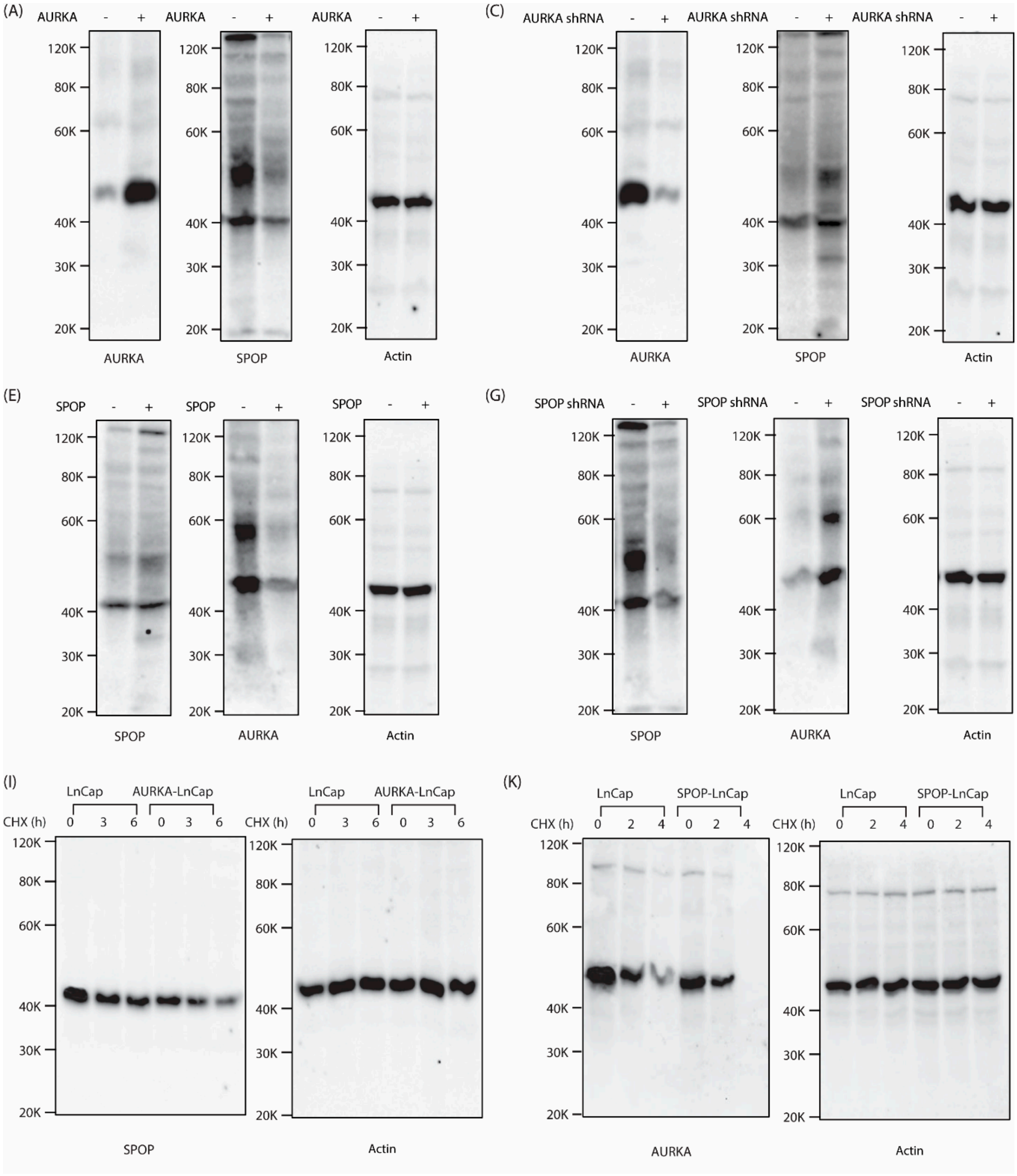

Figure S7. (7A) Full length western blot images of Figure 7A. (7C) Full length western blot images of Figure 7C. (7E) Full length western blot images of Figure 7E. (7G) Full length western blot images of Figure 7G. (7I) Full length western blot images of Figure 7I. (7K) Full length western blot images of Figure 7K.

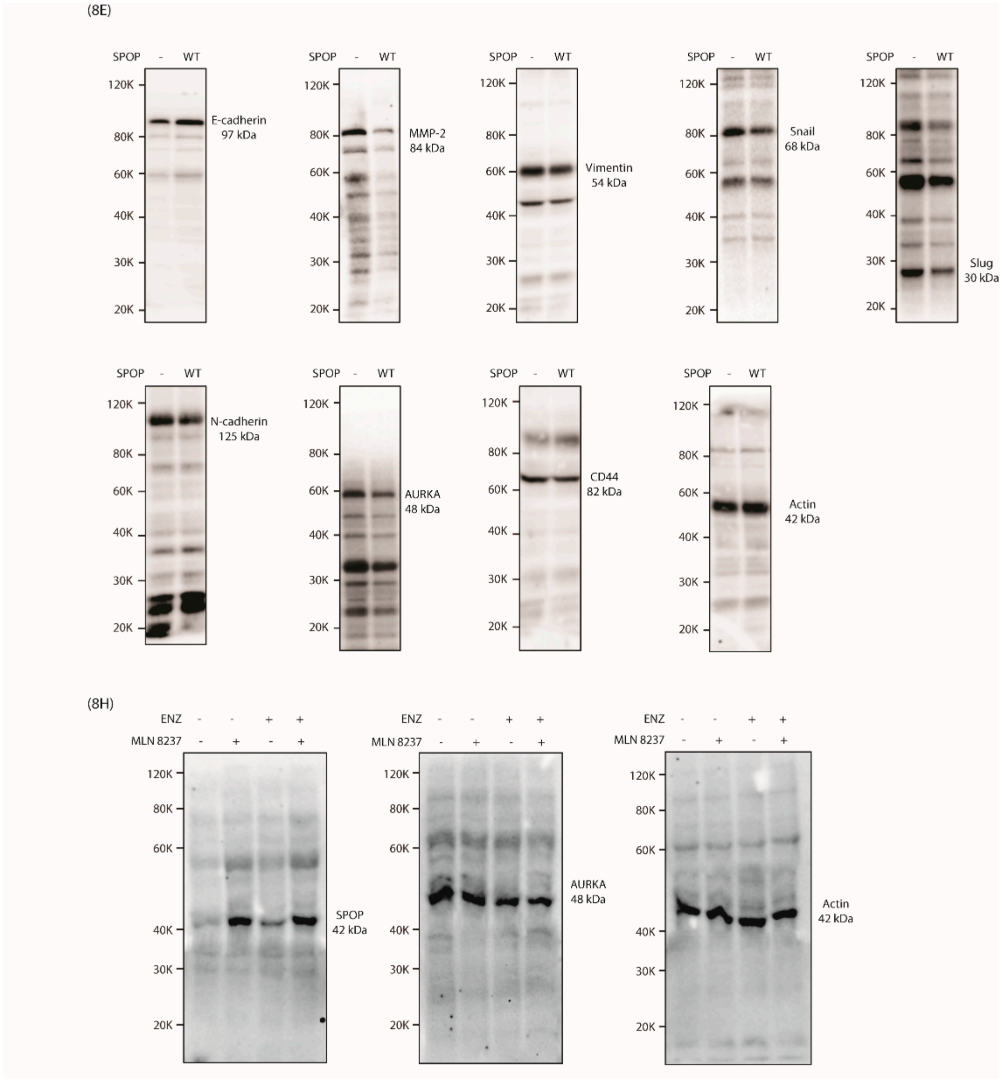

Figure S8. (8E) Full length western blot images of Figure 8E. (8H) Full length western blot images of Figure 8H.

Table S1. List of antibodies used in this study.

	Antibody	Catalogue No.	Company	RRID/Lot
1	AURKA	SC-25425	Santa Cruz Biotech	RRID:AB_2061345
2	Actin	SC-8432	Santa Cruz Biotech	RRID:AB_626630
3	Tubulin	SC-8035	Santa Cruz Biotech	RRID:AB_628408
4	SPOP	SC-377206	Santa Cruz Biotech	
5	SNAIL	PB9439	Boster	Lot#0931512DA859969
6	SLUG	PB9399	Boster	Lot#0931512DA859969
7	N-Cadherin	PA1328	One World Lab	AB_10891323
8	CD44	PA1021	One World Lab	AB_10890445
9	Vimentin	SC-7558	Santa Cruz Biotech	RRID:AB_794002
10	E-Cadherin	bs-10009R	Bioss Inc	Lot#9L18W1
11	MMP-2	bs-4599R	Bioss Inc	RRID:AB_11083963
12	HA	12CA5	Thermo Fisher	RRID:AB_1958069
13	6x-His	AB-1711	Columbia Bioscience	Lot# PUR01016008
14	AR	SC-816	Santa Cruz Biotech	RRID:AB_ 1563391
15	c-Myc	SC-40	Santa Cruz Biotech	RRID:AB_627268
16	Lamin-A	SC-20680	Santa Cruz Biotech	RRID:AB_648148
17	AURKA	14475	Cell Signaling	RRID:AB_2665504

Table S2. Sequence of SPOP shRNA.

SPOP shRNA (forward) CCG GCT CCT ACA TGT GGA CCA TCA ACT CGA GTT GAT GGT CCA CAT GTA GGA GTT TTT TG
SPOP shRNA (reverse) AAT TCA AAA AGA GAA CCC TAA ACC ACA AGA TCT CGA GAT CTT GTG GTT TAG GGT TCT C.

Table S3. Sequences of real time qPCR primers.

Name	Primer Sequences
AURKA F	5'- CCACCTTCGGCATCCTAATA A-3'
AURKA R	5'- TCCAAGTGGTGCATATTCCA -3'
SPOP F	5'- GGAAGGCTCCAAACCTCGACAA-3'
SPOP R	5'- AGCGTTCTCCACGGACAGGTTA-3'
β-Actin F	5'- CATGTACGTTGCTATCCAGGC -3'
β-Actin R	5'- CTCCTTAATGTCACGCACGAT -3'
